# Chronic Social Stress and Immune Dysregulation: Integrating Neuroendocrine Signaling, Microbiota, and Tissue-Specific Responses

**DOI:** 10.3390/cells15151356

**Published:** 2026-07-28

**Authors:** William P. Lafuse, Murugesan V. S. Rajaram

**Affiliations:** Department of Microbial Infection and Immunity, The Ohio State University, Columbus, OH 43210, USA; william.lafuse@osumc.edu

**Keywords:** social disruption stress, glucocorticoids, adrenergic receptors, epinephrine, norepinephrine, macrophage, gut microbiota and lung infection

## Abstract

Psychological stress arises when individuals perceive demands as exceeding their capacity to cope. Social stress can be acute, such as giving a presentation, producing transient increases in heart rate, blood pressure, and neuroendocrine activation, or chronic, in which stressors persist over extended periods. Chronic psychosocial stress disrupts immune homeostasis and contributes to anxiety, depression, and post-traumatic stress, while also increasing risk for infections, inflammatory and autoimmune diseases, cardiovascular disease, metabolic dysfunction, and cancer. Persistent stress engages interconnected neuroendocrine–immune networks, including the hypothalamic–pituitary–adrenal axis, the sympathetic–adrenomedullary system, and the gut microbiota–brain–immune axis, leading to sustained glucocorticoid and catecholamine signaling, gut dysbiosis, impaired barrier integrity, and altered microbial metabolite profiles. In this review, we summarize the molecular and cellular mechanisms by which these axes remodel innate and adaptive immunity and shape disease susceptibility. Using social defeat and social isolation in mice as model systems, we highlight how chronic social stress reprograms immune responses in key tissues, including the lung, brain, and gut.

## 1. Introduction

Psychological stress is a state of mental and physiological tension triggered by challenging or threatening situations. It may be acute, such as during an exam or job interview, or chronic, when stressors persist and exceed an individual’s ability to cope. Common causes of chronic stress include demanding occupations, financial hardship, and adverse social relationships. While acute stress is generally adaptive, chronic stress is harmful because it causes prolonged activation of the stress response. Sustained stress increases the risk of cardiovascular disease, metabolic disorders such as diabetes, and neuropsychiatric conditions, including depression, while also worsening existing diseases.

The physiological response to stress is primarily regulated by the hypothalamic–pituitary–adrenal (HPA) axis, the sympathetic–adrenal–medullary (SAM) axis, and the microbiota–gut–brain–immune axis. The HPA and SAM axes are key regulators of immune function under both homeostatic and stress conditions. Activation of the HPA axis stimulates the release of glucocorticoids (cortisol in humans and corticosterone in rodents), whereas activation of the SAM axis triggers the release of catecholamines, including epinephrine from the adrenal medulla and norepinephrine from sympathetic nerve terminals.

Under homeostatic conditions, glucocorticoids and catecholamines secretion follow a circadian rhythm, peaking during the active phase and declining during the inactive phase. Acute psychological stress rapidly activates both axes, producing the classic “fight or flight” response. Sympathetic activation increases heart rate, dilates airways, and redirects blood flow to skeletal muscles, while HPA activation elevates blood glucose and suppresses non-essential processes such as digestion and certain aspects of immune function. These responses normally return to baseline once the stressor resolves. In contrast, chronic stress leads to persistent activation of the HPA and SAM axes, resulting in long-term neuroendocrine and immune dysregulations.

**Figure 1:**
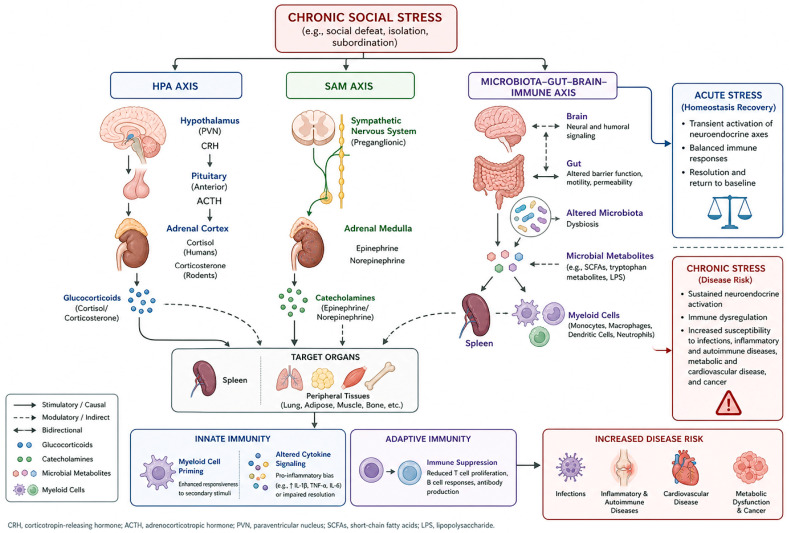
**Chronic social stress engages multiple interconnected pathways that reshape immunity and increase disease risk.** Chronic social stress activates the hypothalamic–pituitary–adrenal (HPA) and sympathetic–adrenal–medullary (SAM) axes, resulting in glucocorticoid and catecholamine release that alters myeloid and lymphocyte function in lymphoid organs and peripheral tissues. Stress also disrupts the microbiota–gut–brain–immune axis by impairing intestinal barrier integrity, inducing dysbiosis, and altering microbial metabolite production. Together, these pathways drive immune dysregulation and increase susceptibility to infections, inflammatory and autoimmune diseases, cardiovascular disease, metabolic disorders, and cancer. Schematic illustration generated with Perplexity (AI assistant); content curated and verified by the authors.

This review examines how the HPA and SAM axes regulate innate and adaptive immunity during homeostasis and chronic stress. We discuss the cellular and molecular mechanisms underlying these effects and how their dysregulation contributes to disease. We also examine the microbiota–gut–brain axis, a bi-directional communication network in which stress-induced sympathetic activation alters gut microbial composition (dysbiosis), while microbial metabolites influence central nervous system function. Emerging evidence suggests that this axis also regulates immune responses during chronic stress by promoting microbial translocation and priming myeloid cells in peripheral tissues such as the spleen (Figure 1).

Finally, we review experimental models of chronic stress, focusing on the mouse social disruption stress (SDR) model. We describe how SDR activates the HPA and SAM axes, alters gut microbiome composition, and affects immune function. We also compare SDR with chronic restraint stress to highlight differences in neuroendocrine, immune, and behavioral outcomes. In addition, we discuss studies of social isolation in adult mice, which induce depression and anxiety-like behaviors together with gut dysbiosis, providing further insight into the interactions among social stress, neuroendocrine signaling, gut microbiota, and immune regulation.

## 2. Hypothalamic–Pituitary–Adrenal Axis

The hypothalamic–pituitary–adrenal (HPA) axis and the sympathetic–adrenal–medullary (SAM) axis are activated in parallel during stress, coordinating the release of glucocorticoids and catecholamines, respectively (Figure 1). HPA activation begins with the secretion of corticotropin-releasing hormone (CRH) by parvocellular neurons in the paraventricular nucleus (PVN) of the hypothalamus [1]. CRH enters the hypophyseal portal circulation and stimulates corticotropes in the anterior pituitary through corticotropin-releasing hormone receptor 1 (CRH1), inducing adrenocorticotropic hormone (ACTH) secretion. ACTH then binds melanocortin type 2 receptor (MC-R2) in the adrenal cortex, stimulating the production of glucocorticoids (cortisol in humans and corticosterone in rodents), which mediate many of the physiological effects of stress (reviewed in [2]). Glucocorticoids regulate metabolism, cardiovascular function, and immunity while providing negative feedback to the hypothalamus and pituitary to terminate HPA axis activation.

### 2.1. Glucocorticoids

Glucocorticoids (GCs) are key regulators of metabolism, brain function, and immune homeostasis (Figure 1). Their secretion follows a circadian rhythm controlled by the suprachiasmatic nucleus through HAP axis activity, autonomic innervation of the adrenal gland, and intrinsic adrenal clocks [3,4]. GCs signal through mineralocorticoids receptors (MR) and glucocorticoid receptors (GR). Because MRs have higher affinity for GCs, they are largely occupied under basal conditions, whereas GRs are preferentially activated during circadian peaks and stress when GC concentrations increase [5,6]. GR is a ligand-activated transcription factor that regulates gene expression by binding glucocorticoid response elements (GREs) or through interactions with other transcription factors. In addition to driving stress adaption, GCs suppress HPA activity through a classical negative-feedback loop [7]. At low GC concentrations, macrophage migration inhibitory factor (MIF) partially counteracts GC-mediated immunosuppression, whereas increasing GC levels suppress MIF expression, allowing stronger anti-inflammatory effects [8,9].

Following stress, circulating GC concentrations rise rapidly, typically peaking within 30 min. GR activation in pituitary corticotrophs inhibits *Pomc* transcription and ACTH release, limiting further HPA activation [6]. Under physiological conditions, GC secretion occurs as ultradian pulses superimposed on the circadian rhythm, with peak levels at the beginning of the active phase (morning in humans and dark in mice) [6].

### 2.2. Regulation of the Immune System by Glucocorticoids

Glucocorticoids are among the most potent endogenous regulators of inflammation and immunity (Figure 1). They suppress inflammatory gene expression directly through negative GREs and indirectly by interfering with major inflammatory signaling pathways. Genome-wide analyses have identified negative GREs in more than 1000 conserved mouse and human genes, highlighting the broad transcriptional effects of GCs [10]. A major mechanism of GC action is inhibition of Toll-like receptor (TLR)-induced inflammatory signaling. Activated GR suppresses NF-κβ and AP-1. GCs dampen TLR signaling via three main mechanisms. First, GC-activated GR directly interacts with NF-κβ and AP-1 activity through direct protein–protein interactions [11,12], induces IkB to retain NF-kB in the cytoplasm [13], and upregulates dual-specificity phosphatase-1 (DUPS1), which inhibits MAPK signaling and reduces expression of pro-inflammatory mediators, including TNF-α, IL-1, and cyclooxygenase 2 [14]. GCs also induce glucocorticoid-induced leucine zipper (GILZ), an important anti-inflammatory protein that inhibits NF-κB, AP-1, Ras, and Raf signaling, thereby suppressing inflammatory responses in multiple immune cell types [15,16]. In addition, GCs induce tristetraprolin (TTP), an RNA-binding protein that promotes degradation of pro-inflammatory cytokine mRNAs [17]. Together, these transcriptional and post-transcriptional mechanisms broadly limit inflammatory responses.

Although predominantly immunosuppressive, GCs can also enhance immune responsiveness by increasing expression of selected cytokine receptors. For example, GCs upregulate IL-2Rα and IL-7Rα on T cells, promoting IL-2- and IL-7-dependent signaling, T-cell survival, and homeostasis despite reduced cytokine production [18,19,20]. Thus, although GCs inhibit TCR-induced signaling and cytokine expression, GC-mediated upregulation of IL-7Ra on T cells promotes T-cell survival by inhibiting apoptosis and enhances T-cell responsiveness to Il-2 through increased IL-2Ra expression driven by IL-7R signaling. Because IL-7 supports the survival of both naive and memory T cells and is essential for maintaining T-cell homeostasis, GCs may also contribute to sustaining immune responses to foreign antigens. GCs also regulate circadian trafficking of naïve T cells by inducing CXCR4 expression through mineralocorticoid receptor signaling, promoting migration into secondary lymphoid organs [21]. The dominant effects of GCs on adaptive immunity are suppression of T-cell activation and effector differentiation. GR signaling inhibits proximal T-cell receptor signaling by suppressing Lck and Fyn activity [22] and induces the inhibitory receptor PD-1 through a GRE within *dcd1* locus [23]. GCs further inhibit Th1 differentiation by suppressing IL-12 production and T-bet activity while promoting Th2-associated cytokines such as IL-4, IL-10, and IL-13 [24]. GILZ contributes substantially to these effects by inhibiting NF-κB and MAPK signaling, suppressing Th1 and Th17 differentiation, and promoting regulatory T-cell development through enhanced TGF-β signaling [16].

Overall, glucocorticoids regulate both innate and adaptive immunity through coordinated effects on inflammatory gene expression, cytokine signaling, immune cell trafficking, and T-cell differentiation (Figure 1). Under homeostatic conditions, circadian GC secretion generates daily rhythms in immune (reviewed in [25]). During chronic stress, sustained HPA activation disrupts these rhythms, resulting in prolonged GC exposure. The impact of chronic GC elevation on immune function depends on stress intensity and duration, circulating hormone concentrations, and the glucocorticoid sensitivity of individual immune cell populations.

## 3. Sympathetic–Adrenal–Medullary Axis

The sympathetic–adrenal–medullary (SAM) axis mediates the rapid physiological response to stress. The sympathetic nervous system (SNS) consists of preganglionic and postganglionic neurons. Preganglionic neurons originate in the thoracic spinal cord and project through the splanchnic nerve to the adrenal medulla, where acetylcholine stimulates chromaffin cells to release the catecholamines epinephrine and norepinephrine into the circulation (Figure 1). These hormones trigger the classic “fight-or-flight” response by increasing heart rate, cardiac output, blood pressure and blood glucose, while redirecting blood flow to skeletal muscles [26]. In parallel, preganglionic neurons synapse with postganglionic neurons in sympathetic ganglia. Postganglionic fibers innervate most peripheral tissues, including primary and secondary lymphoid organs, where they release norepinephrine from axonal varicosities. Norepinephrine acts on adrenergic receptors expressed by immune and stromal cells providing a direct neural pathway through which the brain regulates peripheral immune function [26,27]. Under homeostatic conditions, sympathetic activity follows a circadian rhythm regulated by the suprachiasmatic nucleus, with higher activity during the active phase. Acute stress produces a rapid but transient activation of the SAM axis that promotes physiological adaptation. In contrast, chronic sympathetic activation disrupts immune homeostasis and contributes to chronic inflammation, anxiety, depression, and other stress-associated disorders [28,29].

## 4. SNS Innervation of Lymphoid Tissue

SNS innervation of lymphoid tissue provides a major pathway through which the brain regulates immune function. Sympathetic nerve fibers densely innervate primary and secondary lymphoid organs, whereby norepinephrine is released onto stromal and immune cells. Through adrenergic receptors, norepinephrine regulates hematopoiesis, leukocyte trafficking, cytokine production, and the balance between innate and adaptive immunity.

### 4.1. Bone Marrow

**Figure 2:**
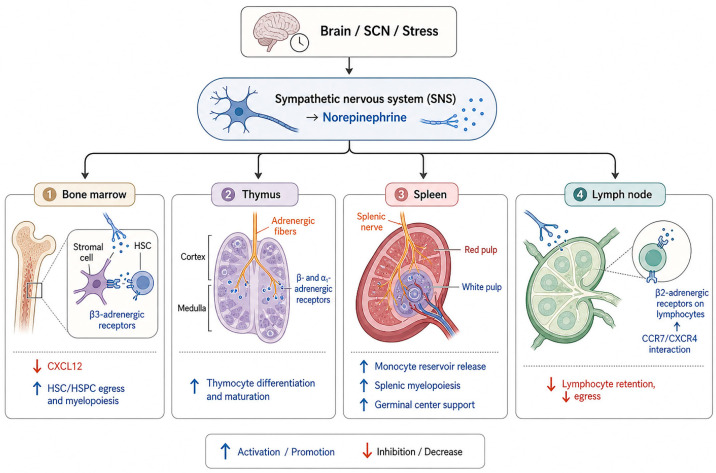
**SNS innervation of lymphoid tissues.** Sympathetic nervous system (SNS) activation triggers norepinephrine release in primary and secondary lymphoid organs to regulate immune homeostasis. In the bone marrow, β3-adrenergic signaling reduces CXCL12 expression, promoting HSC/HSPC egress and myelopoiesis. In the thymus, adrenergic signaling regulates thymocyte differentiation and maturation. In the spleen, sympathetic fibers control monocyte mobilization, splenic myelopoiesis, and germinal center responses. In lymph nodes, β2-adrenergic signaling enhances CCR7/CXCR4-dependent lymphocyte retention and limits egress. Blue/teal symbols indicate activation or promotion, whereas red/orange symbols indicate inhibition or reduction. Schematic illustration generated with Perplexity (AI assistant); content curated and verified by the authors.

The bone marrow is the primary site of hematopoiesis, where hematopoietic stem cells (HSCs) reside within specialized stromal and endothelial niches that support their self-renewal and differentiation into all blood cell lineages [30,31,32,33,34,35,36] (Figure 2). Under homeostatic conditions, sympathetic signaling regulates HSC proliferation and differentiation and trafficking in a circadian manner [36]. Daily oscillations in norepinephrine release control the retention and mobilization of HSCs and hematopoietic progenitor cells (HSPCs) by regulating stromal expression of the chemokine CXCL12, the principal ligand for CXCR4 on HSCs. Activation of β3-adrenergic receptors on stromal cells suppresses CXCL12 expression, promoting HSC and HSPC egress into the circulation, whereas reduced sympathetic tone restores CXCL12 expression and favors their retention in the bone marrow [37,38] (Figure 2).

During infection, HSCs and HSPCs rapidly shift from homeostatic to emergency hematopoiesis. These cells express Toll-like receptors (TLRs), allowing them to directly sense pathogen-associated molecular patterns (PAMPS) [39,40] and increase myeloid cell production (reviewed in [41]). TLR4 signaling promotes HCS proliferation, myeloid differentiation, and mobilization to extramedullary sites such as the spleen, thereby enhancing innate immune responses [42,43,44,45]. Similar adaptations occur during chronic infections. For example, *Leishmania donovani* infection induces HSC expansion and biases myeloid differentiation toward progenitors that are more permissive to parasite survival [46].

Chronic psychological stress similarly reprograms hematopoiesis through sustained sympathetic activation. Increased norepinephrine release activates β3-adrenegic receptors on bone marrow stromal cells, reducing CXCL12 expression and driving HSC proliferation and mobilization [47]. The resulting increase in circulating monocytes and neutrophils contributes to chronic inflammatory diseases, particularly atherosclerosis. In *Apoe* ^−/−^ mice, chronic stress accelerates the development of inflammatory atherosclerotic plaques enriched in myeloid cells, increasing the risk of plaque instability, myocardial infarction, and stroke [48]. Following myocardial infarction, sympathetic signaling further promotes HSC migration to the spleen, where extramedullary hematopoiesis increases monocyte production and recruitment to atherosclerotic lesions, exacerbating disease progression [49].

### 4.2. Thymus

The thymus is the primary lymphoid organ responsible for T-cell development. Bone-marrow-derived progenitors undergo T-cell lineage commitment, T-cell receptor (TCR) rearrangement, and positive selection in the cortex before migrating to the medulla, where self-reactive thymocytes are eliminated by negative selection. Mature naïve T cells then exit the thymus and enter the peripheral immune system.

The thymus is extensively innervated by sympathetic nerve fibers that are distributed throughout the cortex and medulla (Figure 2) [50]. Developing thymocytes, thymic epithelial cells (TECs), dendritic cells, and macrophages are closely associated with these nerve fibers, supporting a role for sympathetic signaling in thymic development and function [50]. Immature and mature thymocytes express β-adrenergic receptors, with receptor expression increasing during maturation [51]. TECs also express β1- and β2- adrenergic receptors [52,53]. Pharmacological blockade of β-adrenergic signaling disrupts thymocyte differentiation and maturation, indicating that sympathetic signaling regulates T-cell development both through thymocytes and indirectly through stromal cells [54,55]. In addition, α1-adrenegic receptors are expressed by thymocytes, TECs, and macrophages, and inhibition of α1- adrenergic signaling also impairs thymocyte differentiation [56]. Together, these studies demonstrate that physiological adrenergic signaling contributes to normal thymopoiesis.

In contrast, chronic stress impairs thymic function. Chronic immobilization stress in mice induces thymic atrophy, reduces thymocyte numbers, and increases thymocyte apoptosis, with immature thymocytes being particularly affected [57]. These findings suggest that prolonged sympathetic activation shifts thymic homeostasis from supporting T-cell development toward reduced thymic output, thereby compromising adaptive immunity.

### 4.3. Spleen and Lymph Nodes

The spleen filters blood removes blood-borne pathogens, and senescent erythrocytes and serves as an important site for innate and adaptive immune responses (reviewed in [58]). It is organized into red pulp and the marginal zone (Figure 2). Macrophages in the red pulp, white pulp, and the marginal zone clear circulating pathogens, while dendritic cells (C^+^DCs) and marginal zone B cells capture antigens and transport them to the white pulp, where T- and B-cell responses are initiated and phagocytose and clear blood-borne pathogens (reviewed in [58,59,60]). The red pulp also functions as a reservoir for Ly6C^hi^ monocytes and supports extramedullary hematopoiesis through hematopoietic stem and progenitor cells (HSPCs) that migrate from the bone marrow under sympathetic control [61]. Sympathetic nerve fibers enter the spleen through the splenic nerve and innervate both the red and white pulp, where they are closely associated with macrophages, DCs, and lymphocyte [62]. In the red pulp, norepinephrine promotes the rapid mobilization of reservoir monocytes during tissue injury and stimulates local myelopoiesis through β-2-adrenergic receptor signaling [63,64]. Thus, sympathetic signaling regulates both the deployment of mature myeloid cells and the generation of new myeloid cells during inflammation.

Within the white pulp, sympathetic signaling also influences adaptive immunity. Germinal centers, where B cells undergo affinity maturation and differentiation into plasma and memory cells, require intact sympathetic innervation. Chemical sympathectomy impairs germinal center formation following *Staphylococcus aureus* infection and reduces protective immunity, although the underlying mechanisms remain incompletely understood [60]. Lymph nodes are similarly innervated by sympathetic fibers that closely associate with DCs, macrophages, and lymphocytes [65]. These organs filter lymph and provide the primary site for antigen presentation and T- and B-cell activation (reviewed in [66,67]). Sympathetic signaling also regulates lymphocyte trafficking. Activation of β-2-adrenergic receptors enhances CCR7- and CXCR4-dependent retention of T and B cells within lymph nodes by functionally interacting with these chemokine receptors [68] (Figure 2). In humans, β-adrenergic blockade reduces T follicular helper (Tfh) cell responses and antigen-specific IgG production following seasonal influenza vaccination, supporting a role for sympathetic signaling in germinal center responses [69]. Together, these findings suggest that chronic sympathetic activation may impair immune surveillance by promoting lymphocyte retention within lymph nodes while simultaneously altering humoral immune responses.

## 5. Adrenergic Receptors

**Figure 3:**
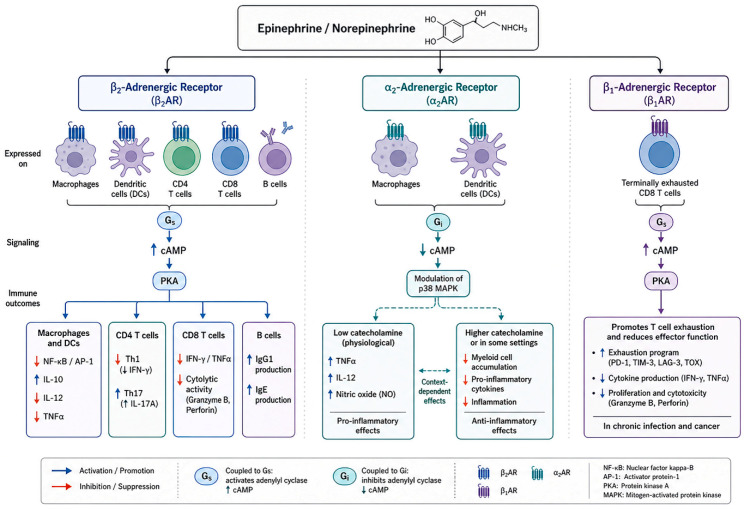
**Adrenergic receptor signaling in immune cells.** Epinephrine and norepinephrine regulate immune cell function through distinct adrenergic receptor subtypes. β_2_-adrenergic receptors activate cAMP/PKA signaling to suppress NF-κB/AP-1-dependent inflammatory responses, increase IL-10 production, inhibit Th1 and CD8 T-cell effector functions, promote Th17 differentiation, and enhance IgG1 and IgE production by B cells. In contrast, α_2_-adrenergic receptors reduce cAMP signaling and can promote antimicrobial and pro-inflammatory responses or suppress inflammation, depending on the cellular context. β_1_-adrenergic receptors contribute to CD8 T-cell exhaustion during chronic infection and cancer, whereas β-blockade restores CD8 T-cell function and improves immunotherapy responses. Blue/teal symbols indicate activation or promotion, and red/orange symbols indicate inhibition or suppression. Gs and Gi denote stimulatory and inhibitory G-protein coupling, respectively. Schematic illustration generated with Perplexity (AI assistant); content curated and verified by the authors.

Epinephrine and norepinephrine signal through five G-protein-coupled adrenergic receptor (AR) subtypes: α1, α2, β1, β2, and β3 (Figure 3). β-ARs primarily couple to G_s_, proteins, stimulating adenylate cyclase and increasing cAMP. In contrast, α2-ARs couple to G_i_ to inhibit cAMP production, whereas α1-ARs couple to G_q_, activating phospholipase C-β, and protein kinase C signaling. Consequently, the effects of catecholamines depend on the repertoire of adrenergic receptors expressed by individual cell types. Among immune cells, the β_2_-adrenergic receptor is the prominent subtype and mediated many of the immunomodulatory effects of sympathetic activation [70].

### 5.1. Adrenergic Receptors and Macrophages

Macrophages express both β_2_- and α_2_-adrenergic receptors, enabling catecholamines to exert context-dependent effects on innate immunity. In general, β_2_-adrenergic receptor signaling suppresses inflammatory responses, whereas α_2_-adrenergic receptor signaling often enhances antimicrobial and pro-inflammatory functions (Figure 3).

Activation of β_2_-adrenergic receptors inhibit the production of TNFα, IL-6, and CCL2 while inducing the anti-inflammatory cytokine IL-10 [71,72,73,74,75]. Increased IL-10 further suppresses macrophage activation by reducing pro-inflammatory cytokine production, MHC class II and CD86 expression, and IL-12 secretion, thereby limiting Th1 polarization [76,77,78,79,80,81]. β_2_-Adrenergic signaling also suppresses macrophage antimicrobial activity by reducing nitric oxide production. Mechanistically, these effects are mediated through the cAMP–protein kinase A (PKA) pathway, which inhibits NF-κB and MAPK signaling by stabilizing IκB and inducing MAPK phosphatases such as MKP1 [82,83,84].

In contrast, α_2_-adrenergic receptor activation frequently promotes inflammatory responses. Stimulation of α_2_-ARs enhances LPS-induced TNFα and IL-12 production and increases macrophage antimicrobial activity through enhanced generation of nitric oxide, superoxide, and peroxynitrite [85,86,87,88]. These effects are associated with G_i_-dependent signaling, inhibition of cAMP production, and activation of p38 MAPK [84]. However, α_2_-adrenergic signaling is not exclusively pro-inflammatory. In some models, α_2_-agonists reduce inflammatory cell recruitment and cytokine production, possibly through inhibition of NF-κB signaling, and can also enhance antigen presentation and anti-tumor immunity [89,90]. These findings indicate that the outcome of α_2_-adrenergic signaling is highly dependent on the inflammatory context.

Because macrophages frequently co-express β_2_- and α_2_-adrenergic receptors, their response to physiological catecholamines reflects the balance between these opposing signaling pathways. We previously showed that low concentrations of epinephrine enhanced macrophage antimicrobial activity, whereas higher concentrations suppressed bacterial killing, suggesting preferential activation of α_2_-ARs at low catecholamine concentrations and β_2_-ARs at higher concentrations [91]. Adrenergic receptor expression is also dynamically regulated during inflammation. Monocytes and macrophages upregulate α_2_-adrenergic receptors following LPS stimulation. β_2_-adrenegic agonists also suppress cytokine gene expression by cAMP-dependent inhibition of the ERK MAP Kinase and cAMP-dependent up-regulation of mitogen-activated protein kinase phosphatase 1 (MKP1), which constrains p38 MAP kinase activity [86], and activated microglia similarly shift from predominantly β_2_- to α_2_-A adrenergic receptor expression [92]. Thus, both catecholamine concentration and inflammatory activation state determine how sympathetic signaling regulates macrophage function.

### 5.2. Adrenergic Receptors and Dendritic Cells

Dendritic cells (DCs) link innate and adaptive immunity by capturing processing and presenting antigens to T cells. DCs express both α- and β-adrenergic receptors [93]. Immature DCs (iDC) express the α_1β_-adrenergic receptors, which promote their migration from inflamed tissues to draining lymph nodes [94], whereas α_2_-adrenergic receptor signaling enhances antigen uptake through PI3K activation [95].

The β_2_-adrenergic receptor is the predominant functional adrenergic receptor on immature DCs [93]. Although β_2_-adrenergic signaling has little effect on DC differentiation or their ability to stimulate naïve T-cell proliferation, it profoundly alters DC cytokine production [93]. β_2_-adrenergic receptor activation suppresses the production of TNFα, IL-6, IL-12, and IL-23 while increasing the anti-inflammatory cytokine IL-10 and the alarmin IL-33 [96,97]. These changes reduce the capacity of DCs to promote Th1 differentiation and IFN-γ production while favoring Th17 responses through altered IL-12/IL-23 signaling [93,98] (Figure 3). Mechanistically, these effects are mediated largely through inhibition of NF-κB and AP-1 signaling downstream of β_2_-adrenergic receptor activation [95].

In addition to modulating effector T-cell responses, β_2_-adrenergic signaling promotes immune regulation by enhancing the ability of DCs to induce Foxp3^+^ regulatory T cells (Tregs) [96]. Moreover, β_2_-adrenergic receptor activation also increases the suppressive activity of Tregs themselves [99]. Collectively, these findings indicate that sympathetic signaling reprograms DC function by altering cytokine production and T-cell polarization, shifting adaptive immunity away from Th1 responses toward Th17 and regulatory pathways.

### 5.3. Adrenergic Receptors and CD4 T Cells

Mouse naïve CD4 T cells and effector Th1 cells express β_2_-adrenergic receptors, whereas effector Th2 cells express little or no β_2_-adrenergic receptor [100,101]. This pattern is established during T-cell differentiation through epigenetic regulation of β_2_-adrenergic receptor locus, including changes in histone acetylation, histone methylation, and DNA methylation [102]. Th1 differentiation is associated with increased β_2_-adrenergic receptor expression, while Th2 differentiation leads to transcriptional repression of the receptor [102].

Functionally, β_2_-adrenergic signaling regulated CD4 T-cell polarization in a cytokine-dependent manner. When naïve CD4 T cells are stimulated in the presence of IL-12, norepinephrine enhances Th1 differentiation and increases IFN-γ production [103]. However, β_2_-adrenergic signaling in DCs and macrophages suppresses IL-12 while promoting IL-23 and IL-6 production [93,104], thereby indirectly limiting Th1 development and favoring Th17 differentiation. Consistent with this indirect effect, β2-adrenergic stimulation reduces Th1 responses and enhances Th17 differentiation in both mice and humans [93,104]. Thus, sustained β_2_-adrenergic signaling shifts the Th1/Th17 balance toward Th17 dominance.

In human peripheral blood mononuclear cells, β_2_-adrenergic agonist decreases IFN-γ production while increasing IL-17A expression through a cAMP/PKA-dependent mechanism [105,106]. In summary, β2-adrenergic stimulation tends to reduce Th1 differentiation and favor Th17 differentiation. Because Th1 cells are critical for immunity against intracellular pathogens such as *Mycobacterium tuberculosis*, whereas Th17 cells promote neutrophil-mediated defense against extracellular bacteria such as *Staphylococcus aureus* [107], chronic sympathetic activation may impair resistance to intracellular infections while enhancing IL-17-driven inflammatory responses. This shift has been implicated in autoimmune and inflammatory diseases, including autoimmune arthritis, psoriatic disease, and asthma [108,109,110].

### 5.4. Adrenergic Receptors and CD8 T Cells

The β_2_-adrenergic receptors regulate multiple aspects of CD8 T-cell function. In humans, β_2_-adrenergic receptor expression is highest on effector memory CD8 T cells and lower on central memory and naïve subsets [111,112,113]. In both mice and humans, norepinephrine suppresses CD8 T-cell effector function by reducing IFN-γ and TNF-α production and impairing cytotoxic activity following T-cell receptor (TCR) stimulation [112,114]. During activation, β_2_-adrenergic signaling also inhibits the metabolic reprograming required for effector differentiation by reducing glucose transporter 1 (GLUT1) expression, glucose uptake, and glycolysis [115]. In memory CD8 T cells, norepinephrine further suppresses IL-2 and IFN-γ production while increasing expression of IL-6 and inflammatory chemokines, resulting in reduced proliferative expansion [113].

Adrenergic signaling also contributes to CD8 T-cell exhaustion during chronic antigen stimulation. Terminally exhausted CD8 T cells selectively upregulate β_1_-adrenergic receptors and are frequently localized near sympathetic nerve fibers in mouse models of chronic viral infection and pancreatic cancer [116] (Figure 3). β_1_-adrenergic receptor overexpression impairs CD8 T-cell proliferation and cytokine production, whereas genetic deletion of β_1_-adrenergic receptors limits the development of terminal exhaustion during chronic infection [116]. These findings identify sympathetic signaling as a direct regulator of T-cell exhaustion.

Consistent with these observations, β-adrenergic signaling suppresses anti-tumor immunity and reduces the efficacy of immunotherapy. In mouse tumor models, β-adrenergic stimulation inhibits CD8 T-cell proliferation, cytokine production, and cytotoxic activity, leading to accelerated tumor growth and reduced responses to immune checkpoint blockade [117]. Conversely, β-blockade enhances vaccine-induced anti-tumor immunity by improving CD8 T-cell priming and increasing tumor infiltration, thereby augmenting the efficacy of checkpoint inhibitors [118,119,120]. Activated tumor-infiltrating CD8 T cells become relatively insensitive to β-adrenergic signaling because they downregulate β_2_-adrenergic receptors, whereas naïve T cells remain highly responsive [120]. Collectively, these studies demonstrate that adrenergic signaling suppresses CD8 T-cell effector function, promotes metabolic dysfunction and terminal exhaustion, and limits anti-tumor and antiviral immunity. Conversely, β-blockade can partially restore CD8 T-cell function and improve responses to immunotherapy.

### 5.5. Adrenergic Receptors and B Cells

B cells predominantly express β_2_-adrenergic receptors, making them direct targets of sympathetic signaling. B-cell activation requires antigen recognition through the B-cell receptor (BCR) together with costimulatory signals provided by CD4+ T cells, including CD40–CD40L interactions and cytokines such as IL-4, IL-5, and IL-6. Activation of β_2_-adrenergic receptors enhances humoral immunity by promoting B-cell proliferation and antibody production [121,122]. β_2_-adrenergic signaling synergizes with BCR activation to increase expression of the costimulatory molecule CD86 through enhanced transcription and mRNA stability [123,124]. Because Th2 cells lack β_2_-adrenergic receptors, catecholamines act directly on B cells to amplify CD86-dependent T-cell help, resulting in increased production of IgG1 and IgE [124]. Mechanistically, β_2_-adrenergic receptor signaling activates the cAMP–PKA pathway, which cooperates with CD86 signaling to enhance transcription of immunoglobulin genes [125,126] (Figure 3).

The functional consequences of β_2_-adrenergic signaling depend on the antibody isotype produced. Increased IgG1 production can enhance protective immunity against bacterial and viral pathogens by promoting opsonization, complement activation, and pathogen neutralization. In contrast, β_2_-adrenergic signaling also augments IgE production through a cAMP/PKA- and p38 MAPK-dependent pathway [127,128,129]. Because IgE is a central mediator of allergic inflammation, sustained sympathetic activation may contribute to allergic diseases such as asthma, allergic rhinitis, and atopic dermatitis by promoting IgE class switching and antibody production [130,131]. Collectively, these findings indicate that sympathetic signaling directly regulates B-cell function by enhancing antibody responses. While this may strengthen protective humoral immunity through increased IgG1 production, chronic β_2_-adrenergic receptor activation may also exacerbate allergic disease by promoting IgE-mediated immune responses.

Collectively, the studies discussed above demonstrate that sympathetic regulation of immunity is highly tissue-specific. Although norepinephrine is the primary neurotransmitter released by sympathetic nerve fibers, its effects vary among organs owing to differences in adrenergic receptor expression, resident immune and stromal cell populations, and the local tissue microenvironment. Consequently, chronic sympathetic activation does not elicit a uniform immunosuppressive or pro-inflammatory response but instead produces distinct patterns of immune regulation across tissues. The major organ-specific effects of sympathetic signaling are summarized in Table 1.

In addition to its direct effects on immune cells through adrenergic signaling, chronic psychological stress also regulates immunity indirectly by altering the gut microbiota and intestinal barrier function. The following section examines the microbiota–gut–brain–immune axis, an interconnected pathway through which stress-induced changes in the gut microbiome influence both systemic immune responses and central nervous system function.

## 6. Microbiota–Gut–Brain–Immune Axis

The microbiota–gut–brain–immune axis is a bidirectional communication network linking the gut microbiota, the central nervous system, and the immune system. Studies in humans and animal models have associated stress-induced alterations in gut microbial composition (dysbiosis) with depression and other stress-related disorders (reviewed in [132]). Stress activates neural and neuroendocrine pathways, including vagal and sympathetic signaling, which alter microbial metabolism and the production of bioactive metabolites.

Among the best-characterized microbial metabolites are the short-chain fatty acids (SCFAs) acetate, propionate, and butyrate, which are generated by bacterial fermentation of dietary fiber. SCFAs enter the circulation and can reach the central nervous system, where they regulate microglial maturation and function through receptors such as FFAR2 [133,134]. Beyond their effects on the brain, SCFAs also influence peripheral immunity. Macrophages and neutrophils express FFAR2, and disruption of SCFA–FFAR2 signaling impairs host defense against bacterial pathogens, including *Klebsiella pneumoniae*, *Citrobacter rodentium*, and *Staphylococcus aureus* (reviewed in [135]). In addition, microbiota-derived tryptophan and bile acid metabolites regulate immune homeostasis by promoting immunoregulatory cell populations while limiting pro-inflammatory responses (reviewed in [136]). Chronic psychological stress also disrupts intestinal barrier integrity, increasing intestinal permeability and facilitating the translocation of commensal bacteria and microbial products into the circulation. We previously demonstrated that chronic social stress induces translocation of *Lactobacillus* species to the spleen, where they prime macrophages and neutrophils and increase IL-1β and IL-23 expression [137]. Similar stress-induced bacterial translocation to secondary lymphoid tissues has also been reported [138].

Under homeostatic conditions, the intestinal mucus layer, epithelial barrier, and mucosal immune system prevent the gut microbiota from entering host tissues [139]. Psychological stress disrupts this barrier in part through corticotropin-releasing factor (CRF), which stimulates mast cell activation and compromises epithelial integrity, allowing bacterial products and microorganisms to cross the intestinal epithelium [140]. Barrier disruption also enhances mucosal immune activation, including increased IL-22 production by Th17 cells. In experimental models, IL-22 acts within the central nervous system to reduce neuronal activation and protect against stress-induced anxiety, highlighting another pathway through which gut immunity influences brain function [141]. Collectively, these findings demonstrate that chronic stress alters microbial composition, intestinal barrier function, and microbial metabolite production, thereby reshaping both central nervous system activity and systemic immunity through the gut microbiota–brain–immune axis.

## 7. Mouse Model of Chronic Stress

Chronic psychological stress is associated with an increased risk of depression, post-traumatic stress disorder (PTSD), cardiovascular disease, diabetes, cancer, and impaired wound healing. These effects are mediated by sustained activation of the HPA and SAM axes, leading to neuroendocrine dysregulation, systemic inflammation, and alterations in the gut microbiota.

### 7.1. Social Disruption Stress (SDR) Model

**Figure 4:**
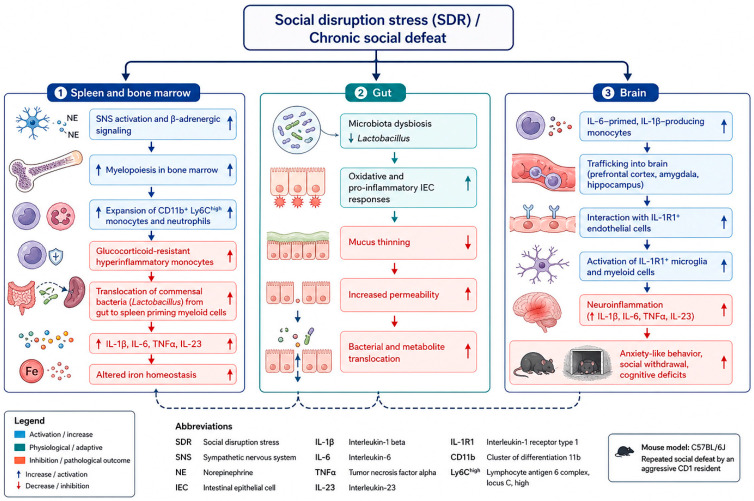
**SDR systemic effects.** Chronic social disruption stress (SDR) activates the HPA axis and sympathetic nervous system, driving coordinated changes in hematopoietic tissues, the gut, and brain. In the spleen and bone marrow, β-adrenergic signaling promotes myelopoiesis, expands glucocorticoid-resistant CD11b^+^Ly6C^high^ monocytes and neutrophils, and, together with microbial translocation, primes myeloid cells toward increased IL-1β, IL-6, TNF-α, and IL-23 production. SDR also disrupts gut homeostasis by inducing dysbiosis, barrier dysfunction, and microbial/metabolite leakage. In the brain, stress-primed inflammatory monocytes infiltrate stress-responsive regions and interact with endothelial cells and microglia, contributing to neuroinflammation and stress-related behavioral changes. Schematic illustration generated with Perplexity (AI assistant); content curated and verified by the authors.

The mouse social disruption stress (SDR) model, also referred to as social defeat stress, is a widely used model of chronic psychosocial stress. In this paradigm, a cage of three resident male mice is exposed to an aggressive CD1 intruder for 2 h daily over six consecutive stress sessions. Repeated social defeat induces anxiety-like behavior, elevates circulating corticosterone, norepinephrine, and epinephrine, increases serum IL-1β and IL-6, and activates myelopoiesis and innate immune responses [142,143] (Figure 4). A related model, chronic social defeat stress (CSDS), extends the duration of social stress. Experimental mice are exposed to an aggressive CD1 mouse for 10 min daily over 10 consecutive days, followed by continuous sensory contact through a perforated divider for the remaining 24 h [142]. This paradigm combines repeated physical and psychological stress and has become a standard model for investigating the behavioral, neuroendocrine, immune, and microbiota-related consequences of chronic stress.

### 7.2. Effects of SDR on Immune Regulation

Social disruption stress (SDR) induces splenomegaly and markedly increases CD11b^+^ monocytes and neutrophils in the spleen through enhanced splenic myelopoiesis [143,144,145]. Elevated monocyte numbers persist for at least 8 days after the final stress cycle, and an expanded CD34^+^ monocyte population remains detectable for up to 24 days, suggesting sustained myeloid activation and the potential for enhanced responses to subsequent stressors [143]. SDR also primes myeloid cells toward a glucocorticoid-resistant, pro-inflammatory phenotype. Monocytes and neutrophils express higher levels of IL-1β, and SDR monocytes produce increased IL-1β, IL-6, and TNF-α following LPS stimulation [137] [146,147]. CD11c^+^ splenic DCs similarly exhibit an activated phenotype, characterized by increased MHC class 1, CD80 and CD44 expression, and enhanced cytokine production [148]. Importantly, these myeloid cells become resistant to glucocorticoid-mediated suppression despite elevated corticosterone levels, an effect that requires IL-1 receptor and TLR4 signaling [145,149]. Because glucocorticoid resistance occurs primarily in wounded mice, it may represent an adaptive mechanism that preserves innate immunity during tissue injury and preserves wound-healing capacity in the face of high corticosterone levels. Consistent with this, the spleen serves as a monocyte reservoir that can rapidly deploy cells to inflamed tissues, including wounds [61]. We propose that sympathetic activation in response to wounding promotes both glucocorticoid resistance and the mobilization of monocytes, as glucocorticoid resistance can be induced via β-adrenergic receptor signaling [150,151] and sympathetic control of β-adrenergic pathways regulate Ly6C^high^ monocytes recruitment to corneal epithelial wounds [63] (Figure 4).

SDR also enhances macrophage antimicrobial activity. Macrophages from stressed mice produce more nitric oxide, superoxide, and peroxynitrite and exhibit greater TLR4-dependent killing of *Escherichia coli* [151,152]. At the molecular level, SDR reprograms monocyte gene expression toward an immature, inflammatory phenotype, with increased expression of genes involved in myeloid differentiation, pattern-recognition receptor signaling, cytokine production, reactive oxygen species generation, and antigen presentation [153,154]. The gut microbiota is required for full expression of this inflammatory phenotype. Germ-free or antibiotic-treated mice fail to develop SDR-induced macrophage priming, whereas microbiota reconstitution restores enhanced antibacterial activity [152]. We further showed that SDR promotes translocation of commensal *Lactobacillus* species to the spleen, where bacterial abundance correlates with increased IL-1β and IL-23 expression, suggesting that microbial translocation contributes to myeloid priming during chronic stress [137].

SDR also reprograms bone marrow hematopoiesis, increasing myelopoiesis while suppressing lymphopoiesis and erythropoiesis through β-adrenergic signaling [155]. Consistent with this, SDR increases hepcidin expression, reduces ferroportin and circulating iron levels, and impairs erythropoiesis, providing a potential mechanism linking chronic psychological stress to anemia [156]. Collectively, these studies demonstrate that SDR induces a persistent glucocorticoid-resistant, inflammatory myeloid phenotype characterized by enhanced myelopoiesis, innate immune priming, and altered hematopoiesis. These changes may improve early antimicrobial defense but also promote chronic inflammation and increase susceptibility to inflammatory and cardiometabolic diseases (Figure 4).

### 7.3. Effects of SDR Stress on Pathogen Infections

Compared with extensive literature examining the effects of SDR on behavior and neuroinflammation, relatively few studies have investigated its impact on host responses to pathogen infections. These studies demonstrate that SDR can either enhance or impair antiviral immunity depending on the pathogen and the balance between innate and adaptive immune responses. In this section, we summarize work on viral infections with influenza A virus (IAV), herpes simplex virus type 1 (HSV-1), and Theiler’s murine encephalomyelitis virus (TMEV), as well as chronic lung infections with *Mycobacterium tuberculosis (M.tb)* and intestinal infection with *Citrobacter rodentium*.

#### 7.3.1. Viral Infections

**Figure 5:**
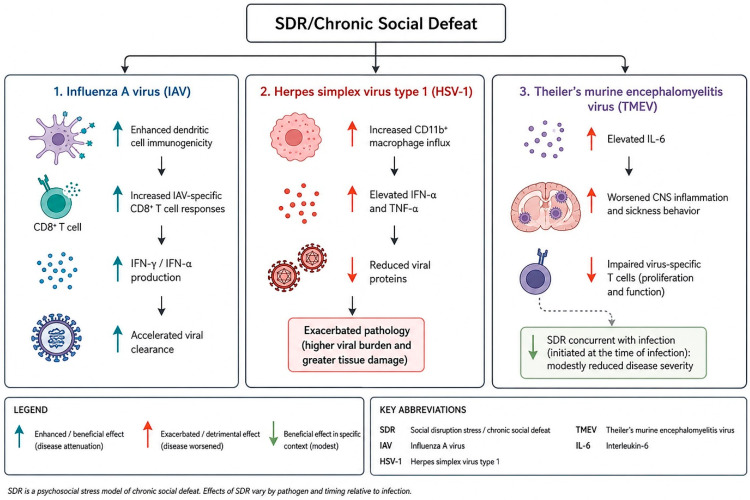
**Chronic social disruption stress (SDR) can enhance or worsen antiviral defenses depending on the pathogen and timing.** Effects of social disruption stress (SDR) on antiviral immunity are pathogen- and context-dependent. Before influenza-A virus (IAV) infection, SDR enhances dendritic cell immunogenicity and IAV-specific CD8^+^ T-cell responses, increasing IFN-γ/IFN-α production and accelerating viral clearance, including during memory responses. Before HSV-1 infection, SDR promoted macrophage recruitment, increased IFN-α and TNF-α expression, and limited viral replication. In contrast, SDR before Theiler’s murine encephalomyelitis virus (TMEV) infection enhances IL-6–driven CNS inflammation, impairs virus-specific CD4^+^ and CD8^+^ T-cell responses, and worsens disease, whereas SDR during infection can reduce disease severity. Blue/teal elements indicate enhanced antiviral responses; red/orange elements indicate impaired immunity or increased pathology. Schematic illustration generated with Perplexity (AI assistant); content curated and verified by the authors.

Among the best-studied examples is influenza A virus (IAV). SDR administered before infection enhances primary antiviral immunity by promoting dendritic cell (DC) activation and increasing virus-specific CD8^+^ T cell response [157,158] (Figure 5). Adoptive transfer experiments showed that DCs isolated from SDR-exposed mice were sufficient to enhance antiviral immunity in naïve recipients, resulting in greater expansion of IAV-specific CD8^+^ T cells and increased antiviral cytokine production following infection [159,160]. SDR also strengthened immunological memory, with stressed mice exhibiting larger pools of memory CD8^+^ T cells and more rapid viral clearance after secondary challenge [160]. These findings indicate that SDR can enhance both primary and memory CD8^+^ T-cell responses to IAV. Similar enhancement of innate immunity has been reported during ocular infection with herpes simplex virus type 1 (HSV-1). SDR increased macrophage recruitment to the cornea and trigeminal ganglia, elevated local expression of IFN-α and TNF-α, and reduced viral replication in nervous tissue, indicating improved early antiviral defense [161,162] (Figure 5). In contrast, SDR exacerbates disease in the mouse model of infection with Theiler’s murine encephalomyelitis virus (TMEV), a model of multiple sclerosis. When SDR was applied before infection, mice developed more severe neurological diseases accompanied by increased IL-6 production, enhanced neuroinflammation, and higher viral loads [163,164]. Neutralization of IL-6 largely prevented these effects, identifying IL-6 as a key mediator of stress-induced disease exacerbation. SDR also impaired virus-specific adaptive immunity by reducing CD4^+^ and CD8^+^ T-cell responses in the central nervous system, despite increased inflammatory responses [165] (Figure 5). Interestingly, SDR applied concurrently with infection reduced disease severity, demonstrating that the timing of stress exposure critically influences disease outcome [166]. Collectively, these studies demonstrate that SDR has pathogen-dependent effects on antiviral immunity. In IAV and HSV-1 infection, SDR enhances innate immune activation and promotes more effective antiviral responses. In contrast, during TMEV infection, SDR increases IL-6-dependent neuroinflammation while impairing virus-specific T-cell immunity, resulting in more severe disease. These findings highlight that the immunological consequences of chronic psychological stress depend on the pathogen, the timing of stress exposure, and the relative contribution of innate versus adaptive immune responses.

#### 7.3.2. Bacterial Infections in the Lung

Tuberculosis remains a major global health threat, causing more than 10 million new cases and approximately 1.2 million deaths annually [167]. Older adults are particularly susceptible to progression from latent to active *Mycobacterium tuberculosis* (*M.tb*) infection, a phenomenon that has been linked to age-associated chronic inflammation (“inflammaging”) [168,169]. Consistent with this concept, aged mice initially control M.tb infection more effectively than young mice but progressively lose control during chronic infection [170,171]. Age-associated changes in alveolar macrophages (AMs) may contribute to this decline in immunity. We previously identified two Am populations, a predominant CD11c^+^ CD11b^−^ resident population with immunoregulatory properties and a smaller CD11c^+^ CD11b^+^ subset of monocytic origin that expands with age and displays a more inflammatory phenotype while supporting greater intracellular *M.tb* growth [172], which suggests that aging remodels the pulmonary macrophage compartment in ways that favor chronic bacterial persistence.

Using the social disruption stress (SDR) model, we next examined how chronic psychological stress alters pulmonary immunity during aging [173]. Unlike the spleen, where SDR primes monocytes for enhanced inflammatory responses, SDR suppressed pulmonary innate immunity, particularly in aged mice. SDR reduced bronchoalveolar levels of IL-1β, TNF-α, and CXCL2 and diminished cytokine responses of alveolar macrophages to TLR2 stimulation, indicating impaired responsiveness to bacterial signals. This immunosuppressive phenotype was accompanied by elevated norepinephrine concentrations in bronchoalveolar lavage fluid, whereas corticosterone levels were unchanged, suggesting that local sympathetic signaling contributes to suppression of pulmonary innate immunity [173]. These changes had important consequences for host defense. SDR did not affect early control of M.tb infection but significantly impaired long-term bacterial control in aged mice. Increased bacterial burden during chronic infection was associated with reduced pulmonary IFN-γ expression, increased IL-10 production, and expansion of IL-10-producing CD4^+^ T cells expressing the type 1 regulatory T-cell (Tr1) markers LAG3 and CD49b. Together, these findings indicate that chronic stress shifts the pulmonary immune response from protective Th1 immunity toward an immunoregulatory phenotype, thereby compromising control of chronic *M.tb* infection.

Emerging evidence also suggests that respiratory infections influence the intestinal microbiota through a gut–lung axis (reviewed in [174,175]). *M.tb* infection alters gut microbial composition within days of infection [176], and respiratory infection with. *Streptococcus pneumoniae* similarly induces gut dysbiosis, particularly in aged mice, where bacterial translocation accompanies increased mortality and impaired bacterial clearance [177]. Although the mechanisms underlying gut–lung communication remain incompletely understood, neural pathways linking pulmonary infection to sympathetic activation may contribute to intestinal barrier dysfunction, microbial translocation, and systemic mobilization of inflammatory myeloid cells. Collectively, these findings demonstrate that chronic psychological stress has tissue-specific effects on innate immunity. Whereas SDR enhances inflammatory responses in peripheral myeloid cells, it suppresses pulmonary immunity in aged mice through increased local sympathetic activity, impaired alveolar macrophage function, and enhanced regulatory T-cell responses, thereby reducing resistance to chronic *M.tb* infection.

#### 7.3.3. Bacterial Infection in the Gut

*Citrobacter rodentium* is a murine colonic pathogen that induces colonic inflammation resembling human inflammatory disease [178]. Infection with *C. rodentium* elicits a Th1-mediated infectious colitis characterized by accumulation of monocytes/macrophages and neutrophils in the colon [178].To determine how SDR affects this model, mice were exposed to SDR during oral challenge of *C. rodentium* [179]. SDR significantly increased colonic pathogen burden and promoted translocation of *C. rodentium* to the spleen, accompanied by elevated colonic macrophages and inflammatory cytokines. These stress-enhanced effects were abolished in CCL2^−/−^mice, demonstrating that CCL2-dependent monocyte recruitment is necessary for SDR-driven exacerbation of colitis [179]. Commensal *Lactobacillus* spp. is known to reduce the severity of *C. rodentium*-induced colitis [180]. Consistent with this, treatment with probiotic *Lactobacillus reuteri* lowered colonic Ccl2 mRNA and attenuated SDR-enhanced colitis, indicating that *L. reuteri* can prevent stress-exacerbated colitis at least in part by downregulating CCL2 [179].

Galley et al. further examined how SDR shapes the colonic mucosa-associated microbiota during *C. rodentium* challenge [181]. Mice were exposed to SDR and then infected with *C. rodentium* or given vehicle control. SDR alone altered the composition of mucosa-associated microbiota; neither pathogen challenge nor vehicle exposure reversed these SDR-induced changes, which persisted for at least 19 days after the last SDR cycle [181]. These findings indicate that SDR induces long-lasting dysbiosis of the mucosal microbiome that is independent of, and not normalized by, subsequent enteric infection [182]. To define the neuroendocrine pathways underlying SDR-enhanced colitis, mice were pretreated with antagonists targeting α_2_- and β-adrenergic (sympathetic) signaling or glucocorticoid and corticotropin-releasing hormone receptor 1 (CRHR1) (HPA axis) signaling prior to SDR and *C. rodentium* infection [183]. SDR upregulated expression of (Duox2), Dual oxidase maturation factor 2 (Duoxa2), and inducible nitric oxide synthase 2 (Nos2) in intestinal epithelial cells (IECs), creating a pro-oxidative, antimicrobial profile; this induction was blocked by β-adrenergic antagonism but not by α_2_-adrenergic, CRH, or glucocorticoid receptor antagonists [183]. SDR also induced microbial dysbiosis that was reversed by β-adrenergic blockade, implicating sympathetic β-adrenergic signaling as a key driver of both epithelial oxidative responses and microbiota disruption [183]. The NADPH oxidase inhibitor apocynin reduced SDR-induced ROS/RNS production, lowered colonic expression of Duox2 and related oxidative enzymes, and diminished the severity of infectious colitis, indicating that SDR sensitizes the gut to inflammation through β-adrenergic-receptor-dependent, NADPH-oxidase-mediated oxidative stress [183].

#### 7.3.4. Effects of SDR Stress on Gut Microbiota and Gut Barrier

The gut microbiota is a largely stable community comprising more than 1000 species that has arisen through ecological successions, favoring microbes best adapted to the intestinal niche [184]. This stability is critical for host health, as abrupt perturbations can result in adverse outcomes such as diarrhea, opportunistic infections, and obesity [185,186]. Galley et al. used high-throughput pyrosequencing to examine the colonic microbiota of C57BL/6 mice exposed to SDR and found that even a single SDR cycle altered the colonic community [187]. SDR did not affect α-diversity but significantly changed β-diversity, indicating shifts in community composition rather than overall richness. At the family level, Porphyromonadaceae were markedly reduced in SDR mice compared with home-cage controls and, at the genus level, Lactobacillus and Parabacteroides were significantly decreased [187]. Quantitative PCR showed that one SDR cycle reduced the relative abundance of Lactobacillus, whereas six cycles were required to significantly lower its absolute abundance, suggesting that SDR effects on the microbiota are cumulative; similar patterns were observed in both inbred C57BL/6 and outbred CD-1 mice [187]. In a related study, the same group reported that the colonic mucosal microbiome differs from the luminal microbiota and that restraint stress reduced *Lactobacillus* spp. in the colonic mucosa but not in the lumen, underscoring compartment-specific stress effects [188].

Our detection of *Lactobacillus* spp. in the spleen of SDR-stressed mice suggests that reduced abundance of Lactobacilli in the colonic mucosa may, at least in part, reflect translocation of these bacteria into the blood circulation and spleen [137]. In addition, sympathetic-nervous-system-driven alterations in gut physiology are likely to influence microbial community structure (reviewed in [189]). Intestinal epithelial cells (IECs) play a significant role in maintaining the physical barrier between microbiota and host, relaying microbial signals to the immune system, and producing factors that shape the gut microbiota [190,191]. Allen et al. examined the impact of SDR on the colonic epithelium in conventionally raised and germ-free mice [192]. RNA sequencing of IECs isolated from conventionally raised SDR mice revealed a pro-inflammatory, pro-oxidative, and antimicrobial transcriptional profile, which coincided with bacterial adhesion to IECs [192]. By contrast, IECs from germ-free SDR mice showed no significant transcriptional changes, indicating that microbiota is required for SDR-induced epithelial reprograming. In conventionally raised mice, SDR increased expression of ROS-generating enzymes such as Duox2 and Nos2 in IECs, paralleling shifts in microbiome composition and suggesting that SDR-induced oxidative stress is a key driver of gut microbiota dysbiosis [192]. In summary, SDR-induced social stress destabilizes the gut microbiota and damages the epithelial barrier, promoting oxidative, pro-inflammatory IEC responses and bacterial translocation, which together create dysbiosis, leaky gut environment that fuels systemic and neuroimmune inflammation (Figure 4).

#### 7.3.5. Effects of SDR Stress on Brain Function

SDR induces anxiety-like behavior that coincides with increased circulation of bone-marrow-derived CD11b^+^/Ly6C^high^ monocytes and accumulation of CD11b^high^ CD45^high^ brain macrophages (Figure 4) [193]. Recruitment of these myeloid cells to the brain is driven by IL-1β and chemokines such as CCL2 and CX3CL1 [194]. SDR increases IL-1β and CCL2 mRNA expression in multiple brain regions, including the cortex, hypothalamus, basal ganglia, and hippocampus [193]. Using LysM-GFP and GFP^+^ bone marrow chimeric mice, Wohleb et al. showed that SDR recruits GFP^+^ macrophages into perivascular spaces and the parenchyma of the prefrontal cortex, amygdala, and hippocampus regions, which are implicated in fear, anxiety, and mood regulation [193]. Mice deficient in chemokine receptors CCR2 and CX3CR1, which are required for monocyte trafficking, failed to recruit monocytes to the brain after SDR and did not develop anxiety-like behavior, indicating that monocyte recruitment to specific brain regions is necessary for the behavioral phenotype and can occur in the absence of classical neuroinflammation [193]. SDR-induced macrophage recruitment and anxiety-like behavior persists for at least 8 days after the final stress cycle but is no longer evident by 24 days. However, a single additional SDR cycle administered 24 days after the initial series triggers renewed translocation of Ly6C^high^ monocytes from the spleen to the brain and re-establishes anxiety-like behavior [144]. For monocytes to enter the brain parenchyma, they must traverse the cerebrovascular endothelium, a process regulated by endothelium adhesion molecules that mediate firm adhesion via integrins on monocytes [195,196]. Intracellular adhesion molecules ICAM-1 and VCAM-1 are key adhesion molecules involved in monocyte–endothelium interactions [197]. Sawicki et al. demonstrated that SDR upregulates VACM-1 and ICAM-1 protein on the vasculature of brain regions that correspond to sites of macrophage trafficking and increases mRNA expression of E-selectin and chemokines CXCL1, CXCL2, and CCL2 in these regions [198]. These changes were localized to CD11b^+^ microglia/macrophages rather than astrocytes, indicating that SDR induces a region-specific neurovascular activation program that facilitates myeloid cell recruitment [198]. Interleukin-1 (IL-1) signaling plays a vital role in SDR-induced neuroinflammation and anxiety-like behavior. Studies using IL-1 receptor type-1 knockout (IL-1R1^−/−^) mice show that IL-1R1 expression is required for SDR-induced increases in circulating monocytes, recruitment of bone-marrow-derived myeloid cells to the brain, and region-specific microglial activation [199,200]. Brain endothelial cells express high levels of IL-1R1 and participate in neuroimmune signaling [201]. Endothelial-specific IL-1R1 knockdown (eIL-1R1kd) preserved SDR-induced monocytosis, monocyte recruitment to the brain, and microglial activation but markedly reduced induction of IL-1β, TNF-α, and IL-6 in brain CD11b^+^ cells and prevented development of anxiety-like behavior [200] (Figure 4). These data indicate that IL-1 signaling in endothelial cells transduces macrophage-derived signals to amplify neuroinflammation and promote anxiety-like behavior. Complementary work using mice with selective deletion of IL-1R1 in glutamatergic neurons of the hippocampus (nIL-1R1^−/−^) showed that neuronal IL-1R1 is required for SDR-induced deficits in social interaction and working memory [202].

RNA sequencing of hippocampal tissue revealed that SDR upregulates canonical inflammatory pathways and upstream regulators in wild-type mice, whereas these changes are abrogated in nL-1R1^−/−^ mice, indicating that IL-1β signaling in neurons drives inflammatory transcriptional programs that modulate social behavior and cognition [202]. Increased IL1R-mediated signaling in neurons after SDR was recently reported to also enhance neuronal reactivity and fear memory [203].

Neurons in the prefrontal cortex, amygdala, and hippocampus are involved in interpretation and response to threatful stimuli induced by SDR. SDR induces unique RNA profiles in projection neurons from the amygdala to the hippocampus and neurons that project from the prefrontal cortex [204]. These unique RNA profiles are associated with hippocampal dependent behavioral and cognitive deficits after SDR.

IL-6 also contributes to SDR-induced monocyte priming and anxiety-like behavior (Figure 4). SDR elevates plasma IL-6 [159]. Niraula et al. examined SDR responses in wild-type and IL-6 knockout mice [160]. SDR-induced anxiety and social avoidance were absent in IL-6 knockout mice, despite normal SD-induced monocyte production in the bone marrow, release into the circulation, and recruitment of CD11b^+^/CD45^high^ monocytes to the brain [160]. Notably, SDR-induced IL-1β expression in the brain was prevented in IL-6 knockout mice, suggesting that IL-6 shapes the activation profile of stress-responsive monocytes. nCounter NanoString analysis showed that blood CD11b^+^Ly6C^high^ monocytes from SDR-exposed wild-type mice exhibited a primed transcriptional profile, which was attenuated in IL-6 knockout mice. Monocytes recruited to the brains of SDR wild-type mice displayed increased expression of inflammatory genes (Ager, Alox5, Ccr1, Cxcl2, Il1b, Mmp8, Myd88, and Stat3), a signature that was absent in IL-6-deficient mice [160]. These findings indicate that IL-6 induced by SDR generates a primed monocyte phenotype that propagates IL-1-mediated neuroinflammation and anxiety and highlights two distinct priming pathways; bacterial translocation drives IL-1β-expressing monocytes in the spleen, whereas IL-6 primes monocytes in the bone marrow [160].

Microglia, the resident immune cells of the brain, are critical for SDR-induced monocyte recruitment and anxiety-like behavior. Depleting microglia with the CSF1R antagonist (PLX5622) prior to SDR abolished monocyte recruitment to the brain and prevented anxiety-like behavior [205]. Transcriptomic profiling showed that SDR-activated microglia up-regulated CCl2, which recruits IL-1β-expressing monocytes; these monocytes then adhere to IL-1R1^+^ endothelial cells, activating the endothelium, a process blocked by microglial depletion [205]. Goodman et al. used single-cell RNA sequencing of hippocampal cells to examine SDR effects in the presence or absence of microglia [206]. SDR induced distinct microglial transcriptional profiles characterized by cytokine/chemokine signaling, cellular stress responses, and enhanced phagocytosis, which were associated with recruited IL-1β-expressing monocytes, macrophages, and neutrophils [206]. Microglial depletion attenuated the SDR-associated transcriptional changes in leukocytes, endothelia, and astrocytes and prevented SDR-induced social withdrawal and cognitive impairment [206]. Hippocampal neurons exhibited robust responses to SDR that were partly microglia dependent, including stress-induced CREB activation, calcium signaling, and changes in glutamatergic receptor signaling. These findings indicate that SDR-activated microglia orchestrate neuroimmune interactions that drive social avoidance and spatial memory deficits [207]. Together SDR-driven chronic social stress creates a feed-forward neuroimmune loop in which IL-6-primed, IL-1β-producing monocytes, activated microglia, and IL-1R1^+^ endothelial and neuronal networks cooperate to generate region-specific neuroinflammation that manifests as anxiety-like behavior, social withdrawal, and cognitive impairment (Figure 4).

Epigenetic regulation of gene expression by chronic stress remains largely unexplored. However, two recent studies have examined histone modifications in mice exposed to chronic social defeat stress (CSDS). Na li et al. [208] reported increased expression of histone deacetylase 5 (HDAC5) and elevated H4 acetylation in the hippocampus of C57BL/6 mice following CSDS. Notably, hippocampal knockdown of HDAC5 reversed CSDS-induced social avoidance and behavioral despair, whereas HDAC5 overexpression increased susceptibility to depression-like behaviors, including social avoidance. These findings identify hippocampal HDAC5 as a key mediator of chronic stress-induced depressive behaviors.

A second study [209] investigated the role of histone H3 lysine 27 mono-methylation (H3K27me) in chronic stress. The authors found that H3K27me levels were increased in the nucleus accumbens (NAc) of male mice susceptible to both early-life stress (ELS) and CSDS. The NAc is a critical brain region involved in reward processing, motivation, and cognitive function. Increased H3K27me1 accumulated at genes regulating neuronal excitability and was associated with induction of SUZ12, a core component of the Polycomb Repressive Complex 2 (PRC2) that establishes H3K27 methylation patterns. These findings suggest that chronic social stress produces a persistent “chromatin scar” that promotes lifelong vulnerability to subsequent stress. Importantly, these H3K27me1 changes were male-specific and were not observed in female mice exposed to CSDS.

The role of microRNAs in regulating gene expression during chronic social stress is also poorly understood and represents an important area for future investigation. For example, a recent study examined the role of miR-184-3p in regulating the hypothalamic–pituitary–adrenal (HPA) axis [210]. Chronic stress induces hyperactivity of the paraventricular nucleus (PVN), resulting in excessive release of corticotropin-releasing hormone (CRH), a key contributor to the development of depression. CRH expression is regulated by cAMP response element-binding protein-regulated transcription coactivator 1 (CRTC1) in the PVN. The study demonstrated that miR-184-3p suppresses CRTC1 expression. CSDS induced depressive-like behaviors while reducing miR-184-3p expression in the PVN. Moreover, genetic knockdown of miR-184-3p in the PVN of naïve mice induced depressive-like behaviors, whereas overexpression of miR-184-3p significantly attenuated the behavioral effects of CSDS. These findings identify miR-184-3p as a potential regulator of stress susceptibility through modulation of HPA axis activity.

#### 7.3.6. Effects of SDR Stress on Autoimmune Diseases

Patients with stress-related disorders frequently exhibit comorbid autoimmune conditions, suggesting that chronic stress can promote or exacerbate autoimmunity. Shimo et al. investigated the relationship between chronic social stress and brain-directed autoimmunity by analyzing antibody responses in chronic social defeat stress (CSDS) mice and in patients with major depressive disorder (MDD) [211]. In CSDS-exposed mice, serum antibody concentrations were elevated, and T and B cell populations were expanded in the cervical lymph nodes that drain the brain. Sera from these socially defeated mice displayed increased reactivity against brain tissue, and levels of brain-reactive IgG positively correlated with the degree of social avoidance. Similarly, sera from MDD patients contained higher levels of brain-reactive antibodies, which correlated with anhedonia. These findings support a link between chronic psychosocial stress, the development of brain-directed autoantibodies, and stress-related behavioral symptoms, and suggest that stress-induced immune dysregulation may contribute to autoimmune processes targeting the central nervous system.

### 7.4. Chronic Restrain Stress

A widely used mouse model of psychological stress is restraint stress, in which mice are placed in well-ventilated 50 mL tubes. To induce chronic stress, mice typically undergo 2–6 h of daily restraint for 7–21 days. This paradigm activates the HPA axis, producing high circulating corticosterone, and only raises splenic norepinephrine after prolonged exposure (21 days) [212]. Consequently, its effects on the immune system differ markedly from those of SDR. Rather than inducing splenomegaly, chronic restraint stress reduces spleen size and increases glucocorticoid receptors expression in the spleen. During 7 days of restraint, splenic immune composition shifts, with increased CD3^+^CD8^+^ T cells and decreased red pulp C11b^+^ F4/80^+^ macrophages and decreased CD11b^+^Ly6G^−^Ly-6C^high^ monocytic myeloid cells; these changes are prevented by the GR antagonist RU486, indicating mediation via HPA activation [212]. In contrast to SDR, which enhances myelopoiesis and suppresses erythropoiesis, chronic restraint stress promotes stress erythropoiesis in the spleen, accompanied by reduced blood iron and elevated circulating erythropoietin and increased splenic c-Kit and BMP4 expression that drives BMP4-dependent erythropoiesis [213].

### 7.5. Social Isolation Stress

Social isolation is another important psychological stressor. In humans, it reflects a lack of social connections (e.g., absence of spouse or limited contact with family and friends) and is more common in older adults, contributing to increased morbidity and mortality via higher risk of cardiovascular disease, hypertension, infections, and cognitive decline. In mice, social isolation is modeled by single housing and compared with group housing. Here we focus on isolation initiated in adulthood. Magalhães et al. showed that 3 weeks of social isolation in middle-aged male C57BL/6J mice induced depressive-like and anxiety-like behaviors and impaired spatial memory [214]. Another study found that 12 weeks of social isolation in middle-aged BALB/c mice produced depressive-like behavior and elevated plasma IL-6 and IL-1β [215]. Gut microbiota were also analyzed in this study by high-throughput sequencing of 16s RNA, which revealed significant changes in β-diversity and a reduced Firmicutes-to-Bacteroidetes ratio, a pattern also reported in patients with major depressive disorder. Socially isolated mice showed expansion of disease-associated families (*Staphylococcaceae*, *Corynebacteriaceae, Moraxellaceae, Aerococcaceae*, and *Alcaligenaceae*) and reductions in putatively beneficial taxa such as *Ruminococcaceae, Akkermansiaceae, and Christensenellaceae*. These findings suggest that chronic social isolation promotes overgrowth of potentially pathogenic bacteria and loss of probiotic populations, alongside changes in microbial metabolites that may influence the nervous system. Notably, responses to social isolation are sex-dependent: aged female mice isolated for 1 month did not develop depressive-like behavior or changes in IL-6, TNF-α, and IL-1β or hippocampal microglial activation but instead displayed increased hyperactivity and exploratory behavior [216]. Few studies have examined immune function in adult socially isolated mice. A recent report showed that social isolation promotes tumor progression [217]. Male C57BL/6J mice and BALB/c mice isolated 7 days before subcutaneous tumor cell injection exhibited accelerated tumor growth and reduced survival relative to group-housed controls. In a B16-F10 lung metastasis model, socially isolated mice developed more rapid metastasis and increased tumor migration from spleen to liver. Single-cell RNA sequencing of tumor-infiltrating CD45^+^ cells revealed a shift in CD8 T cell subsets, with more exhausted CD8^+^ T cells and fewer cytotoxic and memory CD8^+^ T cells in isolated mice, accompanied by reduced CD8^+^ anti-tumor activity. Pharmacological blockade of β_2_-adrenergic signaling reversed this CD8T cell dysfunction, indicating that social isolation impairs CD8 T-cell-mediated anti-tumor immunity via sympathetic nervous system activation of β_2_-adrenergic receptors.

### 7.6. Resilient Versus Susceptible Phenotypes of Chronic Stress

Chronic psychosocial stress does not produce uniform biological outcomes; instead, individuals display variable susceptibility or resilience depending on the magnitude and regulation of neuroendocrine, immune, and microbial responses. In mouse models of chronic social stress, including social disruption stress (SDR) and chronic social defeat stress (CSDS), a subset of animals develops persistent anxiety-like behavior, neuroinflammation, and immune dysregulation, whereas others exhibit limited behavioral and inflammatory changes despite equivalent stress exposure.

Stress-susceptible phenotypes are characterized by sustained sympathetic nervous system activation, enhanced myelopoiesis, and expansion of inflammatory myeloid populations. SDR increases splenic CD11b^+^ monocytes and neutrophils through enhanced myelopoiesis [143,144,145,218], generating Ly6C^high^ monocytes with increased IL-1β expression and enhanced production of IL-1β, IL-6, and TNF-α after stimulation [137,146,147]. Chronic stress also induces glucocorticoid resistance in inflammatory myeloid cells through IL-1R1- and TLR4-dependent mechanisms, allowing persistent immune activation despite elevated corticosterone levels [145,149]. Transcriptomic and epigenetic remodeling further promotes a pro-inflammatory myeloid state, with SDR altering thousands of monocyte genes involved in inflammatory signaling, oxidative metabolism, antigen presentation, and myeloid differentiation [153,154].

Microbiota-dependent immune priming is an additional feature of stress susceptibility. SDR disrupts intestinal microbial composition, reduces Lactobacillus populations, and alters gut barrier function [187,188]. Microbiota are required for full expression of SDR-induced immune activation, as germ-free or antibiotic-treated mice fail to develop enhanced microbicidal activity after stress [152]. Stress-induced intestinal barrier disruption promotes bacterial translocation to peripheral immune organs, where microbial signals contribute to IL-1β-producing myeloid cell activation [137].

Peripheral immune activation contributes directly to stress-induced behavioral abnormalities through neuroimmune communication. SDR promotes recruitment of CD11b^+^Ly6C^high^ monocytes to stress-sensitive brain regions through IL-1β, CCL2, and CX3CL1 signaling [193,194]. This process requires chemokine receptor pathways, as CCR2- and CX3CR1-deficient mice fail to develop monocyte recruitment and anxiety-like behavior [193]. IL-1R1 signaling in endothelial cells and neurons further amplifies neuroinflammation and contributes to anxiety, social withdrawal, and cognitive impairment [200,202]. IL-6 also promotes stress susceptibility by inducing inflammatory programing of circulating monocytes and enabling IL-1β-mediated neuroimmune activation [160].

Two recent studies have investigated mechanisms underlying resilience to chronic social defeat stress (CSDS). The first study [219] demonstrated that active exploratory and defensive behaviors triggered coordinated activation of medial prefrontal cortex (mPFC) neurons and corticotropin-releasing factor (CRF) signaling, promoting resilience to subsequent stress exposure. These findings suggest that adaptive behavioral responses during stress actively engage neural circuits that enhance stress resilience.

The second study [220] examined the role of circadian rhythms in the response to CSDS. Resilience was associated with increased circadian transcriptional oscillations in the hypothalamus, hippocampus, and liver, as well as enhanced coordination of circadian gene expression between the brain and liver. In contrast, stress-susceptible mice exhibited disrupted cross-tissue transcriptional coordination, suggesting that synchronized circadian regulation across central and peripheral tissues may be an important determinant of resilience to chronic social stress.

Stress-resilient phenotypes may also appear to reflect effective regulation of inflammatory responses rather than an absence of stress-induced activation. Resilience may involve preservation of glucocorticoid sensitivity, balanced hematopoiesis, maintenance of intestinal barrier integrity, and reduced recruitment or activation of inflammatory myeloid cells. Regulation of microglial responses may represent an additional checkpoint, as microglial activation is required for SDR-induced monocyte recruitment, neuroinflammatory gene expression, and behavioral deficits [205,206].

Together, these findings support a model in which stress susceptibility results from persistent sympathetic activation, myeloid reprograming, microbiota-driven immune priming, and neuroimmune amplification, whereas resilience reflects the capacity to contain these responses and restore homeostasis. Understanding the mechanisms that determine resilient versus susceptible phenotypes may provide new strategies to prevent stress-associated inflammatory disorders, including depression, cardiovascular disease, metabolic disease, autoimmune disorders, and impaired tissue repair.

### 7.7. Sex Differences in Chronic Social Stress

Women have a higher prevalence of stress-related psychiatric disorders, including major depressive disorder and post-traumatic stress disorder, suggesting that responses to chronic social stress may differ between sexes (reviewed in [221]). However, most social defeat and social disruption stress studies have been performed in male mice because male mice do not typically display aggression toward females. To overcome this limitation, a DREADD-based approach was used to activate the ventrolateral ventromedial hypothalamus of male aggressor mice, enabling them to attack female mice [222]. Female mice exposed to this stress paradigm developed anxiety-like behavior, increased splenic and brain myelopoiesis, and monocyte accumulation, responses that were comparable to those observed in male mice [223].

A separate model induced aggression toward female mice by exposing them to male urine before chronic social defeat stress (CSDS). After 21 days of stress, female mice exhibited anxiety-like behavior and physiological stress responses; however, unlike male mice, they did not develop generalized social avoidance [224]. A recent study [225] reported that CSDS reduced microbiome diversity and caused gut dysbiosis in both male and female mice. CDS altered the abundance of every gut bacterium phylum. Genera in the Firmicutes phylum were reduced in both males and females, but fewer genera were reduced in females. These findings suggest that, although females exhibit neuroimmune responses and gut dysbiosis in response to social stress, behavioral outcomes may differ from those observed in males. Identifying the molecular basis for sexual differences in behavioral outcomes is an area for future research. One possible area of research is determining if differences in epigenetic regulation of brain gene expression between males and females (discussed in Section 7.3.6) influences behavior.

A major limitation of social defeat models is that they primarily capture physical stress rather than emotional stress, which is a major contributor to human depression and stress-related disorders. Emotional stressors, such as loss of social bonds, are difficult to model in rodents. One approach is vicarious social defeat stress, in which mice experience stress by observing aggression toward another mouse. In this paradigm, an observer mouse is housed adjacent to an aggressor–target pair and exposed to repeated social defeat episodes through a transparent divider. Vicarious social defeat induces depression-like behaviors in female mice and provides a model to investigate emotional stress responses (reviewed in [226]). Recent studies in male mice suggest that neuroinflammatory signaling involving ventral tegmental area dopaminergic neurons may contribute to stress-induced social avoidance [226]. Further studies are needed to define sex-specific molecular mechanisms underlying emotional stress responses and their effects on neuroimmune regulation.

Although SDR, restraint stress, and social isolation are all widely used models of chronic psychological stress, they produce distinct neuroendocrine, immune, and behavioral responses. The major similarities and differences among these models are summarized in Table 2.

## 8. Conclusions

Taken together, these models highlight that different forms of psychological stress engage distinct neuroendocrine pathways, HPA-dominant restraint stress, SNS/β-adrenergic-dominant SDR stress and social isolation, thereby imprinting qualitatively different immune phenotypes that can either bias toward myeloid inflammation, stress erythropoiesis, or impaired CD8 T-cell-mediated tumor surveillance. This review concludes that chronic psychosocial stress exerts profound and highly context-dependent effects on the immune system by differentially engaging the HPA axis and sympathetic nervous system, reshaping hematopoiesis, barrier function, microbiota, and tissue-specific immunity. Across models such as social disruption stress, restraint stress, and social isolation, stress hormones and catecholamines reprogram myeloid and lymphoid compartments, driving glucocorticoid-resistant, hyperinflammatory monocytes in some settings, stress erythropoiesis, or iron restriction in others, and impaired CD8 T-cell-mediated tumor surveillance in yet others. The gut–brain–immune axis and organ-specific microbiomes emerge as critical amplifiers of these effects; stress-induced dysbiosis, epithelial oxidative stress, and barrier breakdown foster bacterial translocation and metabolite signaling that prime myeloid cells and shape T cell differentiation, ultimately feeding forward into neuroinflammation, colitis, and altered control of pulmonary and enteric infections. Together, these findings underscore that the health consequences of chronic stress reflect a dynamic interplay between neuroendocrine circuits, immune effector pathways, and microbiota, and they highlight β-adrenergic and IL-1/IL-6-dependent signaling, as well as probiotic and barrier-protective strategies, as promising targets to mitigate stress-driven susceptibility to infection, autoimmunity, allergy, and neuropsychiatric disease.

## 9. Future Directions

Future studies should move beyond single-axis and single-organ readouts to map the integrated spatial and temporal dynamics of chronic social stress across HPA, SAM, and microbiota–gut–brain–immune networks at organismal scale. Systems-level approaches combining longitudinal multi-omics (transcriptomics, metabolomics, and microbiomics), high-parameter immunophenotyping, and circuit-level neuromodulation in social defeat and social isolation models will be critical to define causal pathways from stressor characteristics to specific immune and disease phenotypes. A major priority is to dissect resilience versus susceptibility, including sex, age, and genetic influences, and to identify microbiota- and β-adrenergic-dependent mechanisms that can be targeted to restore barrier integrity, normalize myeloid and lymphoid function, and improve control of infection, autoimmunity, cardiovascular disease, and cancer under chronic stress. Translation of these mechanistic insights into preventive and therapeutic strategies—ranging from stress-modulating behavioral interventions to microbiota-directed and neuroimmune drugs—represents an important next step for reducing the health burden of chronic psychosocial stress.

## Figures and Tables

**Table 1:** Comparison of sympathetic regulation of immune function across lymphoid and peripheral organs during chronic stress.

Organ	Major SNS Target Cells	Effect of Chronic Stress/SNS Activation	Functional Outcome
Bone marrow	Stromal cells, HSCs/HSPCs	β3-AR signaling reduces CXCL12, mobilizes HSCs/HSPCs, increases myelopoiesis	Increased circulating monocytes and neutrophils; promotes inflammatory diseases such as atherosclerosis
Thymus	Thymocytes, thymic epithelial cells	Sustained adrenergic signaling induces thymic atrophy and thymocyte apoptosis	Reduced naïve T-cell output and impaired adaptive immunity
Spleen	Monocytes, macrophages, lymphocytes	Mobilizes monocyte reservoir; enhances extramedullary myelopoiesis; supports germinal center formation	Rapid innate responses but increased inflammatory monocyte recruitment during chronic stress
Lymph nodes	B cells, T cells, DCs	β2-AR signaling enhances lymphocyte retention through CCR7/CXCR4	Reduced lymphocyte recirculation and impaired immune surveillance
Lung	Alveolar macrophages, CD4 T cells	Increased local norepinephrine suppresses macrophage cytokine responses and promotes IL-10-producing Tr1 cells	Impaired control of chronic *M. tuberculosis* infection
Gut	Enteric neurons, epithelial cells, immune cells	Increased permeability, dysbiosis, bacterial translocation	Systemic inflammation, myeloid priming, altered microbiota-derived metabolite signaling
Brain	Microglia, neurons	Microbial metabolites (SCFAs) and cytokines regulate microglial activation; chronic stress promotes neuroinflammation	Anxiety, depression, altered behavior

Chronic psychological stress activates the sympathetic nervous system, leading to distinct neuroimmune responses in different organs. Rather than producing generalized immunosuppression, sympathetic signaling exerts organ-dependent effects on hematopoiesis, lymphocyte development and trafficking, innate immune activation, epithelial barrier integrity, and microbial homeostasis. These tissue-specific responses contribute to diverse outcomes ranging from enhanced inflammation and cardiovascular disease to impaired host defense against chronic infections.

**Table 2:** Comparison of immune and neuroendocrine responses in mouse models of chronic psychological stress.

Feature	Social Disruption Stress (SDR)	Chronic Restraint Stress	Social Isolation
Primary stressor	Repeated social defeat/subordination by aggressive intruder	Repeated physical restraint and loss of movement	Prolonged absence of social interaction
Major stress pathways	Strong SNS activation; increased norepinephrine and epinephrine; HPA activation with elevated corticosterone	Prominent HPA activation; sustained corticosterone elevation; SNS activation varies by duration	HPA and SNS dysregulation; altered stress reactivity
HPA axis	↑ Corticosterone; may promote glucocorticoid resistance in myeloid cells	Strong and sustained corticosterone response; glucocorticoid-mediated immune suppression	Altered circadian HPA regulation and stress hormone responses
SAM/SNS axis	Robust activation; ↑ norepinephrine in spleen, bone marrow, and peripheral tissues	Increased sympathetic tone, particularly during acute/repeated restraint	Altered sympathetic signaling associated with anxiety-like behavior
Bone marrow/spleen effects	↑ β-adrenergic signaling → ↓ CXCL12 → HSPC mobilization; ↑ myelopoiesis; expansion of inflammatory monocytes and neutrophils	Variable effects; glucocorticoid-dependent changes in hematopoiesis and leukocyte distribution	Altered immune cell composition; reduced immune surveillance reported in some models
Myeloid cell phenotype	Priming of CD11b^+^Ly6C^high monocytes and neutrophils; ↑ IL-1β, IL-6, TNFα, IL-23 production	Often immunosuppressive; reduced inflammatory cytokine responses depending on duration	Dysregulated macrophage and microglial activation
T-cell effects	Context dependent; altered CD4 T-cell polarization and impaired antiviral/adaptive responses in some infections	Reduced T-cell proliferation and cytokine production through glucocorticoid effects	Reduced T-cell function and altered inflammatory balance
Gut microbiota effects	Dysbiosis; reduced beneficial Lactobacillus; increased microbial translocation; altered metabolites	Altered microbial diversity and intestinal barrier function	Strong effects on microbiota composition and gut–brain signaling
Brain/neuroimmune effects	IL-1β/IL-6–producing monocyte recruitment; microglial activation; neuroinflammation	Glucocorticoid-dependent neuronal and glial changes	Strong effects on anxiety, depression-like behavior, cognition, and neuroinflammation
Behavioral phenotype	Anxiety-like behavior, social withdrawal, depression-like behaviors	Anxiety-like behavior, reduced exploration, stress coping changes	Depression-like behavior, anhedonia, cognitive impairment
Major disease relevance	Cardiovascular disease, infection susceptibility, inflammatory disorders	Stress-related metabolic disease, immune suppression, inflammation	Depression, anxiety, neurodegeneration, metabolic dysfunction

## Data Availability

No new data was created or analyzed in this study. Methods: Schematic illustrations of Figure 1,Figure 2,Figure 3,Figure 4 and Figure 5 were generated using Perplexity, an AI assistant, based on the author’s conceptual design; all scientific content and interpretation were provided and verified by the authors.

## Abbreviations

HPA	Hypothalamic–pituitary–adrenal a1
SAM	Sympathetic–adrenal-medullary axis
SNS	Sympathetic nervous system
HSCS	Hematopoietic stem cells
HSPCS	Hematopoietic progenitor cells;
TECs	Thymic epithelial cells
AR	Adrenergic receptor
SDR	Social disruption stress
CRH	Corticotropin-releasing hormone
PVN	Paraventricular nucleus
ACTH	Adrenocorticotropic hormone
CRH1	Corticotropin-releasing hormone receptor 1
MC-R2	Melanocortin type 2 receptor
GCS	Glucocorticoids
GR	Glucocorticoid receptor
MR	Mineralocorticoids receptor
GRES	Glucocorticoid response elements
MIF	Macrophage migration inhibitory factor
DUPS-1	Dual-specificity phosphatase-1
GLIZ	Glucocorticoid-induced leucine zipper
TTP	Tristetraprolin
GLUT1	Glucose Transporter 1
SCFA	Short-chain fatty acids
PTSD	Post-traumatic stress disorder
TMEV	Theiler’s murine encephalomyelitis virus
Duox2	Dual oxidase 2
Duoxa2	Dual oxidase maturation factor 2

## References

[1] Vale W., Spiess J., Rivier C., Rivier J. (1981). Characterization of a 41-residue ovine hypothalamic peptide that stimulates secretion of corticotropin and beta-endorphin. Science.

[2] Burford N.G., Webster N.A., Cruz-Topete D. (2017). Hypothalamic-Pituitary-Adrenal Axis Modulation of Glucocorticoids in the Cardiovascular System. Int. J. Mol. Sci..

[3] Nicolaides N.C., Charmandari E., Kino T., Chrousos G.P. (2017). Stress-Related and Circadian Secretion and Target Tissue Actions of Glucocorticoids: Impact on Health. Front. Endocrinol..

[4] Leliavski A., Dumbell R., Ott V., Oster H. (2015). Adrenal clocks and the role of adrenal hormones in the regulation of circadian physiology. J. Biol. Rhythms.

[5] de Kloet E.R. (2014). From receptor balance to rational glucocorticoid therapy. Endocrinology.

[6] Olejniczak I., Oster H., Ray D.W. (2022). Glucocorticoid circadian rhythms in immune function. Semin. Immunopathol..

[7] Uchoa E.T., Aguilera G., Herman J.P., Fiedler J.L., Deak T., de Sousa M.B. (2014). Novel aspects of glucocorticoid actions. J. Neuroendocrinol..

[8] Calandra T., Bernhagen J., Metz C.N., Spiegel L.A., Bacher M., Donnelly T., Cerami A., Bucala R. (1995). MIF as a glucocorticoid-induced modulator of cytokine production. Nature.

[9] Calandra T., Bucala R. (1995). Macrophage migration inhibitory factor: A counter-regulator of glucocorticoid action and critical mediator of septic shock. J. Inflamm..

[10] Surjit M., Ganti K.P., Mukherji A., Ye T., Hua G., Metzger D., Li M., Chambon P. (2011). Widespread negative response elements mediate direct repression by agonist-liganded glucocorticoid receptor. Cell.

[11] Scheinman R.I., Gualberto A., Jewell C.M., Cidlowski J.A., Baldwin A.S. (1995). Characterization of mechanisms involved in transrepression of NF-kappa B by activated glucocorticoid receptors. Mol. Cell. Biol..

[12] Jonat C., Rahmsdorf H.J., Park K.K., Cato A.C., Gebel S., Ponta H., Herrlich P. (1990). Antitumor promotion and antiinflammation: Down-modulation of AP-1 (Fos/Jun) activity by glucocorticoid hormone. Cell.

[13] Auphan N., DiDonato J.A., Rosette C., Helmberg A., Karin M. (1995). Immunosuppression by glucocorticoids: Inhibition of NF-kappa B activity through induction of I kappa B synthesis. Science.

[14] Abraham S.M., Lawrence T., Kleiman A., Warden P., Medghalchi M., Tuckermann J., Saklatvala J., Clark A.R. (2006). Antiinflammatory effects of dexamethasone are partly dependent on induction of dual specificity phosphatase 1. J. Exp. Med..

[15] Ayroldi E., Riccardi C. (2009). Glucocorticoid-induced leucine zipper (GILZ): A new important mediator of glucocorticoid action. FASEB J..

[16] Cannarile L., Delfino D.V., Adorisio S., Riccardi C., Ayroldi E. (2019). Implicating the Role of GILZ in Glucocorticoid Modulation of T-Cell Activation. Front. Immunol..

[17] Smoak K., Cidlowski J.A. (2006). Glucocorticoids regulate tristetraprolin synthesis and posttranscriptionally regulate tumor necrosis factor alpha inflammatory signaling. Mol. Cell. Biol..

[18] Wiegers G.J., Labeur M.S., Stec I.E., Klinkert W.E., Holsboer F., Reul J.M. (1995). Glucocorticoids accelerate anti-T cell receptor-induced T cell growth. J. Immunol..

[19] Franchimont D., Galon J., Vacchio M.S., Fan S., Visconti R., Frucht D.M., Geenen V., Chrousos G.P., Ashwell J.D., O’Shea J.J. (2002). Positive effects of glucocorticoids on T cell function by up-regulation of IL-7 receptor alpha. J. Immunol..

[20] Lee H.C., Shibata H., Ogawa S., Maki K., Ikuta K. (2005). Transcriptional regulation of the mouse IL-7 receptor alpha promoter by glucocorticoid receptor. J. Immunol..

[21] Besedovsky L., Linz B., Born J., Lange T. (2014). Mineralocorticoid receptor signaling reduces numbers of circulating human naïve T cells and increases their CD62L, CCR7, and CXCR4 expression. Eur. J. Immunol..

[22] Löwenberg M., Tuynman J., Bilderbeek J., Gaber T., Buttgereit F., van Deventer S., Peppelenbosch M., Hommes D. (2005). Rapid immunosuppressive effects of glucocorticoids mediated through Lck and Fyn. Blood.

[23] Maeda N., Maruhashi T., Sugiura D., Shimizu K., Okazaki I.M., Okazaki T. (2019). Glucocorticoids potentiate the inhibitory capacity of programmed cell death 1 by up-regulating its expression on T cells. J. Biol. Chem..

[24] Liberman A.C., Refojo D., Druker J., Toscano M., Rein T., Holsboer F., Arzt E. (2007). The activated glucocorticoid receptor inhibits the transcription factor T-bet by direct protein-protein interaction. FASEB J..

[25] Shimba A., Ikuta K. (2020). Glucocorticoids Regulate Circadian Rhythm of Innate and Adaptive Immunity. Front. Immunol..

[26] McCorry L.K. (2007). Physiology of the autonomic nervous system. Am. J. Pharm. Educ..

[27] Buijs R.M., Scheer F.A., Kreier F., Yi C., Bos N., Goncharuk V.D., Kalsbeek A. (2006). Organization of circadian functions: Interaction with the body. Prog. Brain Res..

[28] de Diego A.M., Gandía L., García A.G. (2008). A physiological view of the central and peripheral mechanisms that regulate the release of catecholamines at the adrenal medulla. Acta Physiol..

[29] Chhatar S., Lal G. (2021). Role of adrenergic receptor signalling in neuroimmune communication. Curr. Res. Immunol..

[30] Morrison S.J., Scadden D.T. (2014). The bone marrow niche for haematopoietic stem cells. Nature.

[31] Tikhonova A.N., Dolgalev I., Hu H., Sivaraj K.K., Hoxha E., Cuesta-Domínguez Á., Pinho S., Akhmetzyanova I., Gao J., Witkowski M. (2019). The bone marrow microenvironment at single-cell resolution. Nature.

[32] Baccin C., Al-Sabah J., Velten L., Helbling P.M., Grünschläger F., Hernández-Malmierca P., Nombela-Arrieta C., Steinmetz L.M., Trumpp A., Haas S. (2020). Combined single-cell and spatial transcriptomics reveal the molecular, cellular and spatial bone marrow niche organization. Nat. Cell Biol..

[33] Laurenti E., Göttgens B. (2018). From haematopoietic stem cells to complex differentiation landscapes. Nature.

[34] Dykstra B., Kent D., Bowie M., McCaffrey L., Hamilton M., Lyons K., Lee S.J., Brinkman R., Eaves C. (2007). Long-term propagation of distinct hematopoietic differentiation programs in vivo. Cell Stem Cell.

[35] Yamamoto R., Morita Y., Ooehara J., Hamanaka S., Onodera M., Rudolph K.L., Ema H., Nakauchi H. (2013). Clonal analysis unveils self-renewing lineage-restricted progenitors generated directly from hematopoietic stem cells. Cell.

[36] Fielding C., Méndez-Ferrer S. (2020). Neuronal regulation of bone marrow stem cell niches. F1000Research.

[37] Golan K., Kumari A., Kollet O., Khatib-Massalha E., Subramaniam M.D., Ferreira Z.S., Avemaria F., Rzeszotek S., García-García A., Xie S. (2018). Daily Onset of Light and Darkness Differentially Controls Hematopoietic Stem Cell Differentiation and Maintenance. Cell Stem Cell.

[38] Méndez-Ferrer S., Lucas D., Battista M., Frenette P.S. (2008). Haematopoietic stem cell release is regulated by circadian oscillations. Nature.

[39] Iwasaki A., Medzhitov R. (2004). Toll-like receptor control of the adaptive immune responses. Nat. Immunol..

[40] Kawai T., Akira S. (2010). The role of pattern-recognition receptors in innate immunity: Update on Toll-like receptors. Nat. Immunol..

[41] Sioud M. (2020). Microbial sensing by haematopoietic stem and progenitor cells: Vigilance against infections and immune education of myeloid cells. Scand. J. Immunol..

[42] Sasaki Y., Guo Y.M., Goto T., Ubukawa K., Asanuma K., Kobayashi I., Sawada K., Wakui H., Takahashi N. (2021). IL-6 Generated from Human Hematopoietic Stem and Progenitor Cells through TLR4 Signaling Promotes Emergency Granulopoiesis by Regulating Transcription Factor Expression. J. Immunol..

[43] Burberry A., Zeng M.Y., Ding L., Wicks I., Inohara N., Morrison S.J., Núñez G. (2014). Infection mobilizes hematopoietic stem cells through cooperative NOD-like receptor and Toll-like receptor signaling. Cell Host Microbe.

[44] Herman A.C., Monlish D.A., Romine M.P., Bhatt S.T., Zippel S., Schuettpelz L.G. (2016). Systemic TLR2 agonist exposure regulates hematopoietic stem cells via cell-autonomous and cell-non-autonomous mechanisms. Blood Cancer J..

[45] Yáñez A., Hassanzadeh-Kiabi N., Ng M.Y., Megías J., Subramanian A., Liu G.Y., Underhill D.M., Gil M.L., Goodridge H.S. (2013). Detection of a TLR2 agonist by hematopoietic stem and progenitor cells impacts the function of the macrophages they produce. Eur. J. Immunol..

[46] Abidin B.M., Hammami A., Stäger S., Heinonen K.M. (2017). Infection-adapted emergency hematopoiesis promotes visceral leishmaniasis. PLoS Pathog..

[47] Heidt T., Courties G., Dutta P., Sager H.B., Sebas M., Iwamoto Y., Sun Y., Da Silva N., Panizzi P., van der Laan A.M. (2014). Differential contribution of monocytes to heart macrophages in steady-state and after myocardial infarction. Circ. Res..

[48] Moore K.J., Tabas I. (2011). Macrophages in the pathogenesis of atherosclerosis. Cell.

[49] Dutta P., Courties G., Wei Y., Leuschner F., Gorbatov R., Robbins C.S., Iwamoto Y., Thompson B., Carlson A.L., Heidt T. (2012). Myocardial infarction accelerates atherosclerosis. Nature.

[50] Al-Shalan H.A.M., Hu D., Nicholls P.K., Greene W.K., Ma B. (2019). Immunofluorescent characterization of innervation and nerve-immune cell neighborhood in mouse thymus. Cell Tissue Res..

[51] Fuchs B.A., Albright J.W., Albright J.F. (1988). Beta-adrenergic receptors on murine lymphocytes: Density varies with cell maturity and lymphocyte subtype and is decreased after antigen administration. Cell. Immunol..

[52] Kurz B., Feindt J., von Gaudecker B., Kranz A., Loppnow H., Mentlein R. (1997). Beta-adrenoceptor-mediated effects in rat cultured thymic epithelial cells. Br. J. Pharmacol..

[53] von Patay B., Kurz B., Mentlein R. (1999). Effect of transmitters and co-transmitters of the sympathetic nervous system on interleukin-6 synthesis in thymic epithelial cells. Neuroimmunomodulation.

[54] Rauski A., Kosec D., Vidić-Danković B., Plećas-Solarović B., Leposavić G. (2003). Effects of beta-adrenoceptor blockade on the phenotypic characteristics of thymocytes and peripheral blood lymphocytes. Int. J. Neurosci..

[55] Rauski A., Kosec D., Vidić-Danković B., Radojević K., Plećas-Solarović B., Leposavić G. (2003). Thymopoiesis following chronic blockade of beta-adrenoceptors. Immunopharmacol. Immunotoxicol..

[56] Pesić V., Kosec D., Radojević K., Pilipović I., Perisić M., Vidić-Danković B., Leposavić G. (2009). Expression of alpha1-adrenoceptors on thymic cells and their role in fine tuning of thymopoiesis. J. Neuroimmunol..

[57] Domínguez-Gerpe L., Rey-Méndez M. (2001). Alterations induced by chronic stress in lymphocyte subsets of blood and primary and secondary immune organs of mice. BMC Immunol..

[58] Bronte V., Pittet M.J. (2013). The spleen in local and systemic regulation of immunity. Immunity.

[59] Mebius R.E., Kraal G. (2005). Structure and function of the spleen. Nat. Rev. Immunol..

[60] Borges da Silva H., Fonseca R., Pereira R.M., Cassado Ados A., Álvarez J.M., D’Império Lima M.R. (2015). Splenic Macrophage Subsets and Their Function during Blood-Borne Infections. Front. Immunol..

[61] Swirski F.K., Nahrendorf M., Etzrodt M., Wildgruber M., Cortez-Retamozo V., Panizzi P., Figueiredo J.L., Kohler R.H., Chudnovskiy A., Waterman P. (2009). Identification of splenic reservoir monocytes and their deployment to inflammatory sites. Science.

[62] Hu D., Al-Shalan H.A.M., Shi Z., Wang P., Wu Y., Nicholls P.K., Greene W.K., Ma B. (2020). Distribution of nerve fibers and nerve-immune cell association in mouse spleen revealed by immunofluorescent staining. Sci. Rep..

[63] He S., Liu J., Xue Y., Fu T., Li Z. (2023). Sympathetic Nerves Coordinate Corneal Epithelial Wound Healing by Controlling the Mobilization of Ly6Chi Monocytes From the Spleen to the Injured Cornea. Investig. Ophthalmol. Vis. Sci..

[64] Vasamsetti S.B., Florentin J., Coppin E., Stiekema L.C.A., Zheng K.H., Nisar M.U., Sembrat J., Levinthal D.J., Rojas M., Stroes E.S.G. (2018). Sympathetic Neuronal Activation Triggers Myeloid Progenitor Proliferation and Differentiation. Immunity.

[65] Hu D., Nicholls P.K., Claus M., Wu Y., Shi Z., Greene W.K., Ma B. (2019). Immunofluorescence characterization of innervation and nerve-immune cell interactions in mouse lymph nodes. Eur. J. Histochem..

[66] Kirby A.C., Coles M.C., Kaye P.M. (2009). Alveolar macrophages transport pathogens to lung draining lymph nodes. J. Immunol..

[67] Leirião P., del Fresno C., Ardavín C. (2012). Monocytes as effector cells: Activated Ly-6C(high) mouse monocytes migrate to the lymph nodes through the lymph and cross-present antigens to CD8+ T cells. Eur. J. Immunol..

[68] Nakai A., Hayano Y., Furuta F., Noda M., Suzuki K. (2014). Control of lymphocyte egress from lymph nodes through β2-adrenergic receptors. J. Exp. Med..

[69] Vinuesa C.G., Linterman M.A., Yu D., MacLennan I.C. (2016). Follicular Helper T Cells. Annu. Rev. Immunol..

[70] Nance D.M., Sanders V.M. (2007). Autonomic innervation and regulation of the immune system (1987–2007). Brain Behav. Immun..

[71] Haskó G., Szabó C. (1998). Regulation of cytokine and chemokine production by transmitters and co-transmitters of the autonomic nervous system. Biochem. Pharmacol..

[72] Steinberger K.J., Bailey M.T., Gross A.C., Sumner L.A., Voorhees J.L., Crouser N., Curry J.M., Wang Y., DeVries A.C., Marsh C.B. (2020). Stress-induced Norepinephrine Downregulates CCL2 in Macrophages to Suppress Tumor Growth in a Model of Malignant Melanoma. Cancer Prev. Res..

[73] Boomershine C.S., Lafuse W.P., Zwilling B.S. (1999). Beta2-adrenergic receptor stimulation inhibits nitric oxide generation by Mycobacterium avium infected macrophages. J. Neuroimmunol..

[74] Chen L., Boomershine C., Wang T., Lafuse W.P., Zwilling B.S. (1999). Synergistic interaction of catecholamine hormones and Mycobacterium avium results in the induction of interleukin-10 mRNA expression by murine peritoneal macrophages. J. Neuroimmunol..

[75] Ağaç D., Estrada L.D., Maples R., Hooper L.V., Farrar J.D. (2018). The β2-adrenergic receptor controls inflammation by driving rapid IL-10 secretion. Brain Behav. Immun..

[76] de Waal Malefyt R., Abrams J., Bennett B., Figdor C.G., de Vries J.E. (1991). Interleukin 10(IL-10) inhibits cytokine synthesis by human monocytes: An autoregulatory role of IL-10 produced by monocytes. J. Exp. Med..

[77] Creery W.D., Diaz-Mitoma F., Filion L., Kumar A. (1996). Differential modulation of B7-1 and B7-2 isoform expression on human monocytes by cytokines which influence the development of T helper cell phenotype. Eur. J. Immunol..

[78] Willems F., Marchant A., Delville J.P., Gérard C., Delvaux A., Velu T., de Boer M., Goldman M. (1994). Interleukin-10 inhibits B7 and intercellular adhesion molecule-1 expression on human monocytes. Eur. J. Immunol..

[79] Rutz S., Ouyang W. (2016). Regulation of Interleukin-10 Expression. Adv. Exp. Med. Biol..

[80] Fiorentino D.F., Zlotnik A., Mosmann T.R., Howard M., O’Garra A. (1991). IL-10 inhibits cytokine production by activated macrophages. J. Immunol..

[81] D’Andrea A., Aste-Amezaga M., Valiante N.M., Ma X., Kubin M., Trinchieri G. (1993). Interleukin 10 (IL-10) inhibits human lymphocyte interferon gamma-production by suppressing natural killer cell stimulatory factor/IL-12 synthesis in accessory cells. J. Exp. Med..

[82] Farmer P., Pugin J. (2000). beta-adrenergic agonists exert their "anti-inflammatory" effects in monocytic cells through the IkappaB/NF-kappaB pathway. Am. J. Physiol. Lung Cell. Mol. Physiol..

[83] Keränen T., Hömmö T., Moilanen E., Korhonen R. (2017). β(2)-receptor agonists salbutamol and terbutaline attenuated cytokine production by suppressing ERK pathway through cAMP in macrophages. Cytokine.

[84] Keränen T., Hömmö T., Hämäläinen M., Moilanen E., Korhonen R. (2016). Anti-Inflammatory Effects of β2-Receptor Agonists Salbutamol and Terbutaline Are Mediated by MKP-1. PLoS ONE.

[85] Spengler R.N., Allen R.M., Remick D.G., Strieter R.M., Kunkel S.L. (1990). Stimulation of alpha-adrenergic receptor augments the production of macrophage-derived tumor necrosis factor. J. Immunol..

[86] Spengler R.N., Chensue S.W., Giacherio D.A., Blenk N., Kunkel S.L. (1994). Endogenous norepinephrine regulates tumor necrosis factor-alpha production from macrophages in vitro. J. Immunol..

[87] Miles B.A., Lafuse W.P., Zwilling B.S. (1996). Binding of alpha-adrenergic receptors stimulates the anti-mycobacterial activity of murine peritoneal macrophages. J. Neuroimmunol..

[88] Weatherby K.E., Zwilling B.S., Lafuse W.P. (2003). Resistance of macrophages to Mycobacterium avium is induced by alpha2-adrenergic stimulation. Infect. Immun..

[89] Liu Y.W., Chen C.C., Wang J.M., Chang W.C., Huang Y.C., Chung S.Y., Chen B.K., Hung J.J. (2007). Role of transcriptional factors Sp1, c-Rel, and c-Jun in LPS-induced C/EBPdelta gene expression of mouse macrophages. Cell. Mol. Life Sci..

[90] Zhu J., Naulaerts S., Boudhan L., Martin M., Gatto L., Van den Eynde B.J. (2023). Tumour immune rejection triggered by activation of α2-adrenergic receptors. Nature.

[91] Haller T., Völkl H., Deetjen P., Dietl P. (1996). The lysosomal Ca2+ pool in MDCK cells can be released by ins(1,4,5)P3-dependent hormones or thapsigargin but does not activate store-operated Ca2+ entry. Biochem. J..

[92] Hanlon M.M., McGarry T., Marzaioli V., Amaechi S., Song Q., Nagpal S., Veale D.J., Fearon U. (2023). Rheumatoid arthritis macrophages are primed for inflammation and display bioenergetic and functional alterations. Rheumatology.

[93] Takenaka M.C., Araujo L.P., Maricato J.T., Nascimento V.M., Guereschi M.G., Rezende R.M., Quintana F.J., Basso A.S. (2016). Norepinephrine Controls Effector T Cell Differentiation through β2-Adrenergic Receptor-Mediated Inhibition of NF-κB and AP-1 in Dendritic Cells. J. Immunol..

[94] Maestroni G.J. (2000). Dendritic cell migration controlled by alpha 1b-adrenergic receptors. J. Immunol..

[95] Yanagawa Y., Matsumoto M., Togashi H. (2010). Enhanced dendritic cell antigen uptake via alpha2 adrenoceptor-mediated PI3K activation following brief exposure to noradrenaline. J. Immunol..

[96] Nijhuis L.E., Olivier B.J., Dhawan S., Hilbers F.W., Boon L., Wolkers M.C., Samsom J.N., de Jonge W.J. (2014). Adrenergic β2 receptor activation stimulates anti-inflammatory properties of dendritic cells in vitro. PLoS ONE.

[97] Hardman C., Ogg G. (2016). Interleukin-33, friend and foe in type-2 immune responses. Curr. Opin. Immunol..

[98] Manni M., Granstein R.D., Maestroni G. (2011). β2-Adrenergic agonists bias TLR-2 and NOD2 activated dendritic cells towards inducing an IL-17 immune response. Cytokine.

[99] Guereschi M.G., Araujo L.P., Maricato J.T., Takenaka M.C., Nascimento V.M., Vivanco B.C., Reis V.O., Keller A.C., Brum P.C., Basso A.S. (2013). Beta2-adrenergic receptor signaling in CD4+ Foxp3+ regulatory T cells enhances their suppressive function in a PKA-dependent manner. Eur. J. Immunol..

[100] Sanders V.M., Baker R.A., Ramer-Quinn D.S., Kasprowicz D.J., Fuchs B.A., Street N.E. (1997). Differential expression of the beta2-adrenergic receptor by Th1 and Th2 clones: Implications for cytokine production and B cell help. J. Immunol..

[101] Ramer-Quinn D.S., Baker R.A., Sanders V.M. (1997). Activated T helper 1 and T helper 2 cells differentially express the beta-2-adrenergic receptor: A mechanism for selective modulation of T helper 1 cell cytokine production. J. Immunol..

[102] McAlees J.W., Smith L.T., Erbe R.S., Jarjoura D., Ponzio N.M., Sanders V.M. (2011). Epigenetic regulation of beta2-adrenergic receptor expression in T(H)1 and T(H)2 cells. Brain Behav. Immun..

[103] Swanson M.A., Lee W.T., Sanders V.M. (2001). IFN-gamma production by Th1 cells generated from naive CD4+ T cells exposed to norepinephrine. J. Immunol..

[104] Kim B.J., Jones H.P. (2010). Epinephrine-primed murine bone marrow-derived dendritic cells facilitate production of IL-17A and IL-4 but not IFN-γ by CD4+ T cells. Brain Behav. Immun..

[105] Carvajal Gonczi C.M., Tabatabaei Shafiei M., East A., Martire E., Maurice-Ventouris M.H.I., Darlington P.J. (2017). Reciprocal modulation of helper Th1 and Th17 cells by the β2-adrenergic receptor agonist drug terbutaline. FEBS J..

[106] Carvajal Gonczi C.M., Hajiaghayi M., Gholizadeh F., Xavier Soares M.A., Touma F., Lopez Naranjo C., Rios A.J., Pozzebon C., Daigneault T., Burchell-Reyes K. (2023). The β2-adrenergic receptor agonist terbutaline upregulates T helper-17 cells in a protein kinase A-dependent manner. Hum. Immunol..

[107] Ouyang W., Kolls J.K., Zheng Y. (2008). The biological functions of T helper 17 cell effector cytokines in inflammation. Immunity.

[108] Ehrenfeld M., Shoenfeld Y. (2020). Comment on: Thymectomy in patients with myasthenia gravis increases the risk of autoimmune rheumatic diseases: A nationwide cohort study. Rheumatology.

[109] Cole S., Manghera A., Burns L., Barrett J., Yager N., Rhys H., Skelton A., Cole J., Goodyear C.S., Griffiths M. (2023). Differential regulation of IL-17A and IL-17F via STAT5 contributes to psoriatic disease. J. Allergy Clin. Immunol..

[110] Newcomb D.C., Peebles R.S. (2013). Th17-mediated inflammation in asthma. Curr. Opin. Immunol..

[111] Chowdhury F.Z., Ramos H.J., Davis L.S., Forman J., Farrar J.D. (2011). IL-12 selectively programs effector pathways that are stably expressed in human CD8+ effector memory T cells in vivo. Blood.

[112] Estrada L.D., Ağaç D., Farrar J.D. (2016). Sympathetic neural signaling via the β2-adrenergic receptor suppresses T-cell receptor-mediated human and mouse CD8(+) T-cell effector function. Eur. J. Immunol..

[113] Slota C., Shi A., Chen G., Bevans M., Weng N.P. (2015). Norepinephrine preferentially modulates memory CD8 T cell function inducing inflammatory cytokine production and reducing proliferation in response to activation. Brain Behav. Immun..

[114] Zalli A., Bosch J.A., Goodyear O., Riddell N., McGettrick H.M., Moss P., Wallace G.R. (2015). Targeting ß2 adrenergic receptors regulate human T cell function directly and indirectly. Brain Behav. Immun..

[115] Qiao G., Bucsek M.J., Winder N.M., Chen M., Giridharan T., Olejniczak S.H., Hylander B.L., Repasky E.A. (2019). β-Adrenergic signaling blocks murine CD8(+) T-cell metabolic reprogramming during activation: A mechanism for immunosuppression by adrenergic stress. Cancer Immunol. Immunother..

[116] Globig A.M., Zhao S., Roginsky J., Maltez V.I., Guiza J., Avina-Ochoa N., Heeg M., Araujo Hoffmann F., Chaudhary O., Wang J. (2023). The β(1)-adrenergic receptor links sympathetic nerves to T cell exhaustion. Nature.

[117] Nissen M.D., Sloan E.K., Mattarollo S.R. (2018). β-Adrenergic Signaling Impairs Antitumor CD8(+) T-cell Responses to B-cell Lymphoma Immunotherapy. Cancer Immunol. Res..

[118] Vingert B., Adotevi O., Patin D., Jung S., Shrikant P., Freyburger L., Eppolito C., Sapoznikov A., Amessou M., Quintin-Colonna F. (2006). The Shiga toxin B-subunit targets antigen in vivo to dendritic cells and elicits anti-tumor immunity. Eur. J. Immunol..

[119] Thoreau M., Penny H.L., Tan K., Regnier F., Weiss J.M., Lee B., Johannes L., Dransart E., Le Bon A., Abastado J.P. (2015). Vaccine-induced tumor regression requires a dynamic cooperation between T cells and myeloid cells at the tumor site. Oncotarget.

[120] Daher C., Vimeux L., Stoeva R., Peranzoni E., Bismuth G., Wieduwild E., Lucas B., Donnadieu E., Bercovici N., Trautmann A. (2019). Blockade of β-Adrenergic Receptors Improves CD8(+) T-cell Priming and Cancer Vaccine Efficacy. Cancer Immunol. Res..

[121] Kruszewska B., Felten S.Y., Moynihan J.A. (1995). Alterations in cytokine and antibody production following chemical sympathectomy in two strains of mice. J. Immunol..

[122] Kohm A.P., Sanders V.M. (1999). Suppression of antigen-specific Th2 cell-dependent IgM and IgG1 production following norepinephrine depletion in vivo. J. Immunol..

[123] Kohm A.P., Mozaffarian A., Sanders V.M. (2002). B cell receptor- and beta 2-adrenergic receptor-induced regulation of B7-2 (CD86) expression in B cells. J. Immunol..

[124] Kasprowicz D.J., Kohm A.P., Berton M.T., Chruscinski A.J., Sharpe A., Sanders V.M. (2000). Stimulation of the B cell receptor, CD86 (B7-2), and the beta 2-adrenergic receptor intrinsically modulates the level of IgG1 and IgE produced per B cell. J. Immunol..

[125] Podojil J.R., Kin N.W., Sanders V.M. (2004). CD86 and beta2-adrenergic receptor signaling pathways, respectively, increase Oct-2 and OCA-B Expression and binding to the 3’-IgH enhancer in B cells. J. Biol. Chem..

[126] Kin N.W., Sanders V.M. (2006). CD86 stimulation on a B cell activates the phosphatidylinositol 3-kinase/Akt and phospholipase C gamma 2/protein kinase C alpha beta signaling pathways. J. Immunol..

[127] Depue R.A., Woodburn L. (1975). Disappearance of paranoid symptoms with chronicity. J. Abnorm. Psychol..

[128] Pongratz G., McAlees J.W., Conrad D.H., Erbe R.S., Haas K.M., Sanders V.M. (2006). The level of IgE produced by a B cell is regulated by norepinephrine in a p38 MAPK- and CD23-dependent manner. J. Immunol..

[129] McAlees J.W., Sanders V.M. (2009). Hematopoietic protein tyrosine phosphatase mediates beta2-adrenergic receptor-induced regulation of p38 mitogen-activated protein kinase in B lymphocytes. Mol. Cell. Biol..

[130] Kolmus K., Tavernier J., Gerlo S. (2015). β2-Adrenergic receptors in immunity and inflammation: Stressing NF-κB. Brain Behav. Immun..

[131] Sharma D., Farrar J.D. (2020). Adrenergic regulation of immune cell function and inflammation. Semin. Immunopathol..

[132] Chang L., Wei Y., Hashimoto K. (2022). Brain-gut-microbiota axis in depression: A historical overview and future directions. Brain Res. Bull..

[133] O’Riordan K.J., Collins M.K., Moloney G.M., Knox E.G., Aburto M.R., Fülling C., Morley S.J., Clarke G., Schellekens H., Cryan J.F. (2022). Short chain fatty acids: Microbial metabolites for gut-brain axis signalling. Mol. Cell. Endocrinol..

[134] Erny D., Hrabě de Angelis A.L., Jaitin D., Wieghofer P., Staszewski O., David E., Keren-Shaul H., Mahlakoiv T., Jakobshagen K., Buch T. (2015). Host microbiota constantly control maturation and function of microglia in the CNS. Nat. Neurosci..

[135] Schlatterer K., Peschel A., Kretschmer D. (2021). Short-Chain Fatty Acid and FFAR2 Activation—A New Option for Treating Infections?. Front. Cell. Infect. Microbiol..

[136] Wang J., Zhu N., Su X., Gao Y., Yang R. (2023). Gut-Microbiota-Derived Metabolites Maintain Gut and Systemic Immune Homeostasis. Cells.

[137] Lafuse W.P., Gearinger R., Fisher S., Nealer C., Mackos A.R., Bailey M.T. (2017). Exposure to a Social Stressor Induces Translocation of Commensal Lactobacilli to the Spleen and Priming of the Innate Immune System. J. Immunol..

[138] Bailey M.T., Engler H., Sheridan J.F. (2006). Stress induces the translocation of cutaneous and gastrointestinal microflora to secondary lymphoid organs of C57BL/6 mice. J. Neuroimmunol..

[139] Zhuang M., Zhang X., Cai J. (2024). Microbiota-gut-brain axis: Interplay between microbiota, barrier function and lymphatic system. Gut Microbes.

[140] Vanuytsel T., van Wanrooy S., Vanheel H., Vanormelingen C., Verschueren S., Houben E., Salim Rasoel S., Tόth J., Holvoet L., Farré R. (2014). Psychological stress and corticotropin-releasing hormone increase intestinal permeability in humans by a mast cell-dependent mechanism. Gut.

[141] Xia M., Lu J., Lan J., Teng T., Shiao R., Sun H., Jin Z., Liu X., Wang J., Wu H. (2025). Elevated IL-22 as a result of stress-induced gut leakage suppresses septal neuron activation to ameliorate anxiety-like behavior. Immunity.

[142] Golden S.A., Covington H.E., Berton O., Russo S.J. (2011). A standardized protocol for repeated social defeat stress in mice. Nat. Protoc..

[143] Wohleb E.S., McKim D.B., Shea D.T., Powell N.D., Tarr A.J., Sheridan J.F., Godbout J.P. (2014). Re-establishment of anxiety in stress-sensitized mice is caused by monocyte trafficking from the spleen to the brain. Biol. Psychiatry.

[144] McKim D.B., Patterson J.M., Wohleb E.S., Jarrett B.L., Reader B.F., Godbout J.P., Sheridan J.F. (2016). Sympathetic Release of Splenic Monocytes Promotes Recurring Anxiety Following Repeated Social Defeat. Biol. Psychiatry.

[145] Avitsur R., Padgett D.A., Dhabhar F.S., Stark J.L., Kramer K.A., Engler H., Sheridan J.F. (2003). Expression of glucocorticoid resistance following social stress requires a second signal. J. Leukoc. Biol..

[146] Avitsur R., Stark J.L., Dhabhar F.S., Sheridan J.F. (2002). Social stress alters splenocyte phenotype and function. J. Neuroimmunol..

[147] Avitsur R., Kavelaars A., Heijnen C., Sheridan J.F. (2005). Social stress and the regulation of tumor necrosis factor-alpha secretion. Brain Behav. Immun..

[148] Powell N.D., Bailey M.T., Mays J.W., Stiner-Jones L.M., Hanke M.L., Padgett D.A., Sheridan J.F. (2009). Repeated social defeat activates dendritic cells and enhances Toll-like receptor dependent cytokine secretion. Brain Behav. Immun..

[149] Engler H., Bailey M.T., Engler A., Stiner-Jones L.M., Quan N., Sheridan J.F. (2008). Interleukin-1 receptor type 1-deficient mice fail to develop social stress-associated glucocorticoid resistance in the spleen. Psychoneuroendocrinology.

[150] Walsh C.P., Bovbjerg D.H., Marsland A.L. (2021). Glucocorticoid resistance and β2-adrenergic receptor signaling pathways promote peripheral pro-inflammatory conditions associated with chronic psychological stress: A systematic review across species. Neurosci. Biobehav. Rev..

[151] Allen R.G., Lafuse W.P., Galley J.D., Ali M.M., Ahmer B.M., Bailey M.T. (2012). The intestinal microbiota are necessary for stressor-induced enhancement of splenic macrophage microbicidal activity. Brain Behav. Immun..

[152] Allen R.G., Lafuse W.P., Powell N.D., Webster Marketon J.I., Stiner-Jones L.M., Sheridan J.F., Bailey M.T. (2012). Stressor-induced increase in microbicidal activity of splenic macrophages is dependent upon peroxynitrite production. Infect. Immun..

[153] Barrett T.J., Corr E.M., van Solingen C., Schlamp F., Brown E.J., Koelwyn G.J., Lee A.H., Shanley L.C., Spruill T.M., Bozal F. (2021). Chronic stress primes innate immune responses in mice and humans. Cell Rep..

[154] Powell N.D., Sloan E.K., Bailey M.T., Arevalo J.M., Miller G.E., Chen E., Kobor M.S., Reader B.F., Sheridan J.F., Cole S.W. (2013). Social stress up-regulates inflammatory gene expression in the leukocyte transcriptome via β-adrenergic induction of myelopoiesis. Proc. Natl. Acad. Sci. USA.

[155] McKim D.B., Yin W., Wang Y., Cole S.W., Godbout J.P., Sheridan J.F. (2018). Social Stress Mobilizes Hematopoietic Stem Cells to Establish Persistent Splenic Myelopoiesis. Cell Rep..

[156] Kasahara E., Nakamura A., Morimoto K., Ito S., Hori M., Sekiyama A. (2024). Social defeat stress impairs systemic iron metabolism by activating the hepcidin-ferroportin axis. FASEB Bioadv..

[157] Mays J.W., Powell N.D., Hunzeker J.T., Hanke M.L., Bailey M.T., Sheridan J.F. (2012). Stress and the anti-influenza immune response: Repeated social defeat augments clonal expansion of CD8(+)T cells during primary influenza A viral infection. J. Neuroimmunol..

[158] Powell N.D., Mays J.W., Bailey M.T., Hanke M.L., Sheridan J.F. (2011). Immunogenic dendritic cells primed by social defeat enhance adaptive immunity to influenza A virus. Brain Behav. Immun..

[159] Stark J.L., Avitsur R., Hunzeker J., Padgett D.A., Sheridan J.F. (2002). Interleukin-6 and the development of social disruption-induced glucocorticoid resistance. J. Neuroimmunol..

[160] Niraula A., Witcher K.G., Sheridan J.F., Godbout J.P. (2019). Interleukin-6 Induced by Social Stress Promotes a Unique Transcriptional Signature in the Monocytes That Facilitate Anxiety. Biol. Psychiatry.

[161] Cathcart H.M., Zheng M., Covar J.J., Liu Y., Podolsky R., Atherton S.S. (2011). Interferon-gamma, macrophages, and virus spread after HSV-1 injection. Investig. Ophthalmol. Vis. Sci..

[162] Dong-Newsom P., Powell N.D., Bailey M.T., Padgett D.A., Sheridan J.F. (2010). Repeated social stress enhances the innate immune response to a primary HSV-1 infection in the cornea and trigeminal ganglia of Balb/c mice. Brain Behav. Immun..

[163] Oleszak E.L., Chang J.R., Friedman H., Katsetos C.D., Platsoucas C.D. (2004). Theiler’s virus infection: A model for multiple sclerosis. Clin. Microbiol. Rev..

[164] Njenga M.K., Asakura K., Hunter S.F., Wettstein P., Pease L.R., Rodriguez M. (1997). The immune system preferentially clears Theiler’s virus from the gray matter of the central nervous system. J. Virol..

[165] Young E.E., Vichaya E.G., Reusser N.M., Cook J.L., Steelman A.J., Welsh C.J., Meagher M.W. (2013). Chronic social stress impairs virus specific adaptive immunity during acute Theiler’s virus infection. J. Neuroimmunol..

[166] Johnson R.R., Storts R., Welsh T.H., Welsh C.J., Meagher M.W. (2004). Social stress alters the severity of acute Theiler’s virus infection. J. Neuroimmunol..

[167] WHO (2015). WHO Tuberculosis Fact Sheet 2015.

[168] Negin J., Abimbola S., Marais B.J. (2015). Tuberculosis among older adults--time to take notice. Int. J. Infect. Dis..

[169] Ault R., Dwivedi V., Koivisto E., Nagy J., Miller K., Nagendran K., Chalana I., Pan X., Wang S.H., Turner J. (2018). Altered monocyte phenotypes but not impaired peripheral T cell immunity may explain susceptibility of the elderly to develop tuberculosis. Exp. Gerontol..

[170] Orme I. (1995). Mechanisms underlying the increased susceptibility of aged mice to tuberculosis. Nutr. Rev..

[171] Turner J., Frank A.A., Orme I.M. (2002). Old mice express a transient early resistance to pulmonary tuberculosis that is mediated by CD8 T cells. Infect. Immun..

[172] Lafuse W.P., Rajaram M.V.S., Wu Q., Moliva J.I., Torrelles J.B., Turner J., Schlesinger L.S. (2019). Identification of an Increased Alveolar Macrophage Subpopulation in Old Mice That Displays Unique Inflammatory Characteristics and Is Permissive to Mycobacterium tuberculosis Infection. J. Immunol..

[173] Lafuse W.P., Wu Q., Kumar N., Saljoughian N., Sunkum S., Ahumada O.S., Turner J., Rajaram M.V.S. (2022). Psychological stress creates an immune suppressive environment in the lung that increases susceptibility of aged mice to Mycobacterium tuberculosis infection. Front. Cell. Infect. Microbiol..

[174] Enjeti A., Sathkumara H.D., Kupz A. (2023). Impact of the gut-lung axis on tuberculosis susceptibility and progression. Front. Microbiol..

[175] Li R., Li J., Zhou X. (2024). Lung microbiome: New insights into the pathogenesis of respiratory diseases. Signal Transduct. Target. Ther..

[176] Winglee K., Eloe-Fadrosh E., Gupta S., Guo H., Fraser C., Bishai W. (2014). Aerosol Mycobacterium tuberculosis infection causes rapid loss of diversity in gut microbiota. PLoS ONE.

[177] McMahan R.H., Hulsebus H.J., Najarro K.M., Giesy L.E., Frank D.N., Orlicky D.J., Kovacs E.J. (2022). Age-Related Intestinal Dysbiosis and Enrichment of Gut-specific Bacteria in the Lung Are Associated With Increased Susceptibility to Streptococcus pneumoniae Infection in Mice. Front. Aging.

[178] Mundy R., MacDonald T.T., Dougan G., Frankel G., Wiles S. (2005). Citrobacter rodentium of mice and man. Cell. Microbiol..

[179] Mackos A.R., Galley J.D., Eubank T.D., Easterling R.S., Parry N.M., Fox J.G., Lyte M., Bailey M.T. (2016). Social stress-enhanced severity of Citrobacter rodentium-induced colitis is CCL2-dependent and attenuated by probiotic Lactobacillus reuteri. Mucosal Immunol..

[180] Rodrigues D.M., Sousa A.J., Johnson-Henry K.C., Sherman P.M., Gareau M.G. (2012). Probiotics are effective for the prevention and treatment of Citrobacter rodentium-induced colitis in mice. J. Infect. Dis..

[181] Galley J.D., Mackos A.R., Varaljay V.A., Bailey M.T. (2017). Stressor exposure has prolonged effects on colonic microbial community structure in Citrobacter rodentium-challenged mice. Sci. Rep..

[182] Chai Z., Lyu Y., Chen Q., Wei C.H., Snyder L.M., Weaver V., Sebastian A., Albert I., Li Q., Cantorna M.T. (2022). Transcriptional Profiling of the Small Intestine and the Colon Reveals Modulation of Gut Infection with Citrobacter rodentium According to the Vitamin A Status. Nutrients.

[183] Caetano-Silva M.E., Hilt M.E., Valishev I., Lim C., Kasperek M., Shrestha A., Fu H., Eck E., McCusker R., Armstrong H. (2026). Social stress worsens colitis through β-adrenergic-driven oxidative stress in intestinal mucosal compartments. Brain Behav. Immun..

[184] Ley R.E., Peterson D.A., Gordon J.I. (2006). Ecological and evolutionary forces shaping microbial diversity in the human intestine. Cell.

[185] Turnbaugh P.J., Ley R.E., Mahowald M.A., Magrini V., Mardis E.R., Gordon J.I. (2006). An obesity-associated gut microbiome with increased capacity for energy harvest. Nature.

[186] Sekirov I., Tam N.M., Jogova M., Robertson M.L., Li Y., Lupp C., Finlay B.B. (2008). Antibiotic-induced perturbations of the intestinal microbiota alter host susceptibility to enteric infection. Infect. Immun..

[187] Galley J.D., Nelson M.C., Yu Z., Dowd S.E., Walter J., Kumar P.S., Lyte M., Bailey M.T. (2014). Exposure to a social stressor disrupts the community structure of the colonic mucosa-associated microbiota. BMC Microbiol..

[188] Galley J.D., Yu Z., Kumar P., Dowd S.E., Lyte M., Bailey M.T. (2014). The structures of the colonic mucosa-associated and luminal microbial communities are distinct and differentially affected by a prolonged murine stressor. Gut Microbes.

[189] Gur T.L., Bailey M.T. (2016). Effects of Stress on Commensal Microbes and Immune System Activity. Adv. Exp. Med. Biol..

[190] Johansson M.E., Sjövall H., Hansson G.C. (2013). The gastrointestinal mucus system in health and disease. Nat. Rev. Gastroenterol. Hepatol..

[191] Atarashi K., Tanoue T., Ando M., Kamada N., Nagano Y., Narushima S., Suda W., Imaoka A., Setoyama H., Nagamori T. (2015). Th17 Cell Induction by Adhesion of Microbes to Intestinal Epithelial Cells. Cell.

[192] Allen J.M., Mackos A.R., Jaggers R.M., Brewster P.C., Webb M., Lin C.H., Ladaika C., Davies R., White P., Loman B.R. (2022). Psychological stress disrupts intestinal epithelial cell function and mucosal integrity through microbe and host-directed processes. Gut Microbes.

[193] Wohleb E.S., Powell N.D., Godbout J.P., Sheridan J.F. (2013). Stress-induced recruitment of bone marrow-derived monocytes to the brain promotes anxiety-like behavior. J. Neurosci..

[194] Prinz M., Priller J. (2010). Tickets to the brain: Role of CCR2 and CX3CR1 in myeloid cell entry in the CNS. J. Neuroimmunol..

[195] Wilson E.H., Weninger W., Hunter C.A. (2010). Trafficking of immune cells in the central nervous system. J. Clin. Investig..

[196] Greenwood J., Heasman S.J., Alvarez J.I., Prat A., Lyck R., Engelhardt B. (2011). Review: Leucocyte-endothelial cell crosstalk at the blood-brain barrier: A prerequisite for successful immune cell entry to the brain. Neuropathol. Appl. Neurobiol..

[197] Nourshargh S., Hordijk P.L., Sixt M. (2010). Breaching multiple barriers: Leukocyte motility through venular walls and the interstitium. Nat. Rev. Mol. Cell Biol..

[198] Sawicki C.M., McKim D.B., Wohleb E.S., Jarrett B.L., Reader B.F., Norden D.M., Godbout J.P., Sheridan J.F. (2015). Social defeat promotes a reactive endothelium in a brain region-dependent manner with increased expression of key adhesion molecules, selectins and chemokines associated with the recruitment of myeloid cells to the brain. Neuroscience.

[199] Wohleb E.S., Hanke M.L., Corona A.W., Powell N.D., Stiner L.M., Bailey M.T., Nelson R.J., Godbout J.P., Sheridan J.F. (2011). β-Adrenergic receptor antagonism prevents anxiety-like behavior and microglial reactivity induced by repeated social defeat. J. Neurosci..

[200] Wohleb E.S., Patterson J.M., Sharma V., Quan N., Godbout J.P., Sheridan J.F. (2014). Knockdown of interleukin-1 receptor type-1 on endothelial cells attenuated stress-induced neuroinflammation and prevented anxiety-like behavior. J. Neurosci..

[201] Quan N., Banks W.A. (2007). Brain-immune communication pathways. Brain Behav. Immun..

[202] DiSabato D.J., Nemeth D.P., Liu X., Witcher K.G., O’Neil S.M., Oliver B., Bray C.E., Sheridan J.F., Godbout J.P., Quan N. (2021). Interleukin-1 receptor on hippocampal neurons drives social withdrawal and cognitive deficits after chronic social stress. Mol. Psychiatry.

[203] Goodman E.J., Biltz R.G., Packer J.M., DiSabato D.J., Swanson S.P., Oliver B., Quan N., Sheridan J.F., Godbout J.P. (2024). Enhanced fear memory after social defeat in mice is dependent on interleukin-1 receptor signaling in glutamatergic neurons. Mol. Psychiatry.

[204] Biltz R.G., Yin W., Goodman E.J., Wangler L.M., Davis A.C., Oliver B.T., Godbout J.P., Sheridan J.F. (2025). Repeated social defeat in male mice induced unique RNA profiles in projection neurons from the amygdala to the hippocampus. Brain Behav. Immun. Health.

[205] McKim D.B., Weber M.D., Niraula A., Sawicki C.M., Liu X., Jarrett B.L., Ramirez-Chan K., Wang Y., Roeth R.M., Sucaldito A.D. (2018). Microglial recruitment of IL-1β-producing monocytes to brain endothelium causes stress-induced anxiety. Mol. Psychiatry.

[206] Goodman E.J., DiSabato D.J., Sheridan J.F., Godbout J.P. (2024). Novel microglial transcriptional signatures promote social and cognitive deficits following repeated social defeat. Commun. Biol..

[207] Weber M.D., Godbout J.P., Sheridan J.F. (2017). Repeated Social Defeat, Neuroinflammation, and Behavior: Monocytes Carry the Signal. Neuropsychopharmacology.

[208] Li N., Liu T., Wang Y.Y., Xu T., Shi H.J., Chang L., Zhu L.J. (2024). Hippocampal HDAC5-mediated histone acetylation underlies stress susceptibility in mice exposed to chronic social defeat stress. Neuroscience.

[209] Torres-Berrío A., Estill M., Patel V., Ramakrishnan A., Kronman H., Minier-Toribio A., Issler O., Browne C.J., Parise E.M., van der Zee Y.Y. (2024). Mono-methylation of lysine 27 at histone 3 confers lifelong susceptibility to stress. Neuron.

[210] Xu D.W., Li W.Y., Shi T.S., Wang C.N., Zhou S.Y., Liu W., Chen W.J., Zhu B.L., Fei H., Cheng D.D. (2024). MiR-184-3p in the paraventricular nucleus participates in the neurobiology of depression via regulation of the hypothalamus-pituitary-adrenal axis. Neuropharmacology.

[211] Shimo Y., Cathomas F., Lin H.Y., Chan K.L., Parise L.F., Li L., Ferrer-Pérez C., Muhareb S., Costi S., Murrough J.W. (2023). Social stress induces autoimmune responses against the brain. Proc. Natl. Acad. Sci. USA.

[212] Li Y., Jiang W., Li Z.Z., Zhang C., Huang C., Yang J., Kong G.Y., Li Z.F. (2018). Repetitive restraint stress changes spleen immune cell subsets through glucocorticoid receptor or β-adrenergic receptor in a stage dependent manner. Biochem. Biophys. Res. Commun..

[213] Vignjević S., Budeč M., Marković D., Dikić D., Mitrović O., Mojsilović S., Durić S.V., Koko V., Cokić B.B., Cokić V. (2014). Chronic psychological stress activates BMP4-dependent extramedullary erythropoiesis. J. Cell. Mol. Med..

[214] Magalhães D.M., Mampay M., Sebastião A.M., Sheridan G.K., Valente C.A. (2024). Age-related impact of social isolation in mice: Young vs middle-aged. Neurochem. Int..

[215] Ding L., Liu J., Yang Y., Cui Z., Du G. (2024). Chronically socially isolated mice exhibit depressive-like behavior regulated by the gut microbiota. Heliyon.

[216] Sullens D.G., Gilley K., Jensen K., Vichaya E., Dolan S.L., Sekeres M.J. (2021). Social isolation induces hyperactivity and exploration in aged female mice. PLoS ONE.

[217] Zhao X., Li F., Cheng C., Bi M., Li J., Cong J., Wang X. (2025). Social isolation promotes tumor immune evasion via β2-adrenergic receptor. Brain Behav. Immun..

[218] Reale M., Iarlori C., Gambi F., Feliciani C., Salone A., Toma L., DeLuca G., Salvatore M., Conti P., Gambi D. (2004). Treatment with an acetylcholinesterase inhibitor in Alzheimer patients modulates the expression and production of the pro-inflammatory and anti-inflammatory cytokines. J. Neuroimmunol..

[219] Kigar S.L., Lynall M.E., DePuyt A.E., Atkinson R., Sun V.H., Samuels J.D., Eassa N.E., Poffenberger C.N., Lehmann M.L., Listwak S.J. (2025). Chronic social defeat stress induces meningeal neutrophilia via type I interferon signaling in male mice. Nat. Commun..

[220] Savva C., Vlassakev I., Bunney B.G., Bunney W.E., Massier L., Seldin M., Sassone-Corsi P., Petrus P., Sato S. (2024). Resilience to Chronic Stress Is Characterized by Circadian Brain-Liver Coordination. Biol. Psychiatry Glob. Open Sci..

[221] Laman-Maharg A., Trainor B.C. (2017). Stress, sex, and motivated behaviors. J. Neurosci. Res..

[222] Takahashi A., Chung J.R., Zhang S., Zhang H., Grossman Y., Aleyasin H., Flanigan M.E., Pfau M.L., Menard C., Dumitriu D. (2017). Establishment of a repeated social defeat stress model in female mice. Sci. Rep..

[223] Yin W., Gallagher N.R., Sawicki C.M., McKim D.B., Godbout J.P., Sheridan J.F. (2019). Repeated social defeat in female mice induces anxiety-like behavior associated with enhanced myelopoiesis and increased monocyte accumulation in the brain. Brain Behav. Immun..

[224] Iñiguez S.D., Flores-Ramirez F.J., Riggs L.M., Alipio J.B., Garcia-Carachure I., Hernandez M.A., Sanchez D.O., Lobo M.K., Serrano P.A., Braren S.H. (2018). Vicarious Social Defeat Stress Induces Depression-Related Outcomes in Female Mice. Biol. Psychiatry.

[225] Garcia I., Kilic F., Bryan C.A., Castro-Vildosola J., Jonnalagadda S.A., Kasturi A., Tilly J., Smith J., Valentin S., Moncayo S. (2025). Social stress changes gut microbiome composition in male, female, and aggressor mice. Brain Behav. Immun. Health.

[226] Patel S., Kushwaha R., Holkar A., Dhaygude S.S., Kumar A., Chakravarty S. (2025). Modeling vicarious social defeat stress in rodents for investigating emotional stress-induced depression. J. Psychiatr. Res..

